# Machine Learning Optimization of Laser Ablation in Liquid for the Green and Low-Cost Synthesis of Clean Gold Nanoparticles

**DOI:** 10.1021/jacs.6c02047

**Published:** 2026-04-02

**Authors:** Runpeng Miao, Catherine Reffatto, Mattia Cattelan, Rafael Torres-Mendieta, Luca Menilli, Vincenzo Amendola

**Affiliations:** † Department of Chemical Sciences, 9308 University of Padova, via Marzolo 1, Padova I-35131, Italy; ‡ Department of Pharmaceutical and Pharmacological Sciences, University of Padova, via Marzolo 5, Padova I-35131, Italy

## Abstract

While gold nanoparticles (Au NPs) are widely employed in modern technology, their large-scale synthesis still faces challenges related to cost and sustainability. In addition, chemical contaminants are a problem when the highest purity is demanded, such as for biomedicine, catalysis, and several processes mediated by the NP surface. Laser ablation in liquid (LAL) is a promising technique for producing surface-clean Au NPs, although its scalability has not yet matched that of conventional chemical methods. In this work, the LAL synthesis of 5 nm Au NPs in a batch configuration was optimized using machine learning. A 3.4-fold increase in investment-specific productivity was achieved compared to the previous LAL record, and at 1/18 of the initial investment. This makes the laser synthesis of Au NPs “greener” and four times cheaper than gram-scale chemical synthesis via the classical Turkevich–Frens method. Besides, the chemical-free and surface-clean Au NPs showed better cytocompatibility, superior performance as MALDI substrates, higher catalytic activity in the reduction of nitrothiophenol, higher surface thiol coverage, and a more intense plasmon absorption compared to that of the commercial counterpart. This study highlights the positive prospects of machine learning-optimized LAL for the low-cost and environmentally sustainable production of metal NPs possessing convenient properties not achievable through wet-chemistry routes.

## Graphical abstract

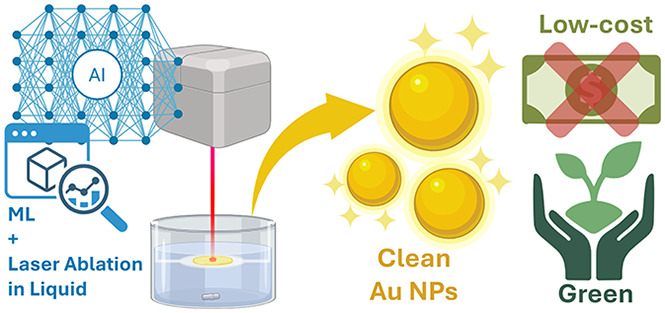

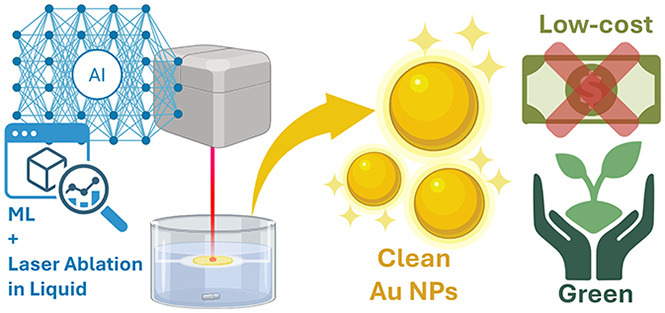

## Introduction

Gold nanoparticles (Au NPs) are among the most common, intensively investigated, and widely exploited nanotechnologies.^1–4^ The U.S. market value of Au NPs exceeds 1 billion USD.^5^ This success is due to their multiple physical and chemical properties, including physicochemical stability, biocompatibility, the presence of localized surface plasmon resonance (LSPR), the combination of chemical nobility with the reactivity of undercoordinated Au atoms at the NP surface, and the preservation of metallic properties down to sizes of a few nanometers.^1–4,6^

However, to meet current industrial trends and requirements, the synthesis of Au NPs requires reducing the cost of the final product while simultaneously increasing the sustainability of the production method.^7,8^ Another critical aspect is the absence of chemical contaminants, both in the solution containing the Au NPs or physisorbed/chemisorbed on the NP surface, because this compromises biocompatibility and cytocompatibility, interferes with the interaction of substrates in catalytic processes, or hampers the binding of functional ligands such as antibodies or self-assembled monolayers with surface gold atoms.^2,7,9–14^

Laser ablation in liquid has been recognized for years as a method capable of producing in a sustainable manner Au NPs with a high degree of purity and biocompatibility.^10,15–19^ LAL consists of the generation of Au NPs by focusing high-fluence laser pulses onto a bulk gold target immersed in a solution of pure water usually containing salt at a low concentration, for example, 100 μM NaCl.^9,15^ Despite the simplicity of the system, which can also be automated and remotely controlled while minimizing operator involvement,^20,21^ the scalability of the synthesis remains a substantial limitation.^10,22,23^ Indeed, there has been extensive research aimed at improving the productivity of LAL-synthesized Au NPs by addressing various factors that impact the synthesis process.^10,22–29^ In parallel, process validation and monitoring protocols were developed to meet the Food and Drug Administration (FDA) and Quality-by-Design (QbD) frameworks.^21^ The current record for mass productivity (MP) of Au NPs in water is 3.8 g/h, obtained with ultrafast (<5 ps), high-power (>100 W) lasers with fast scanning speeds (>10 m/s).^24,25^ However, this is an expensive setup, resulting in an initial investment exceeding 500 k€ and an investment-specific productivity (ISP, given by the ratio of MP to the setup cost)^22,30^ of 5.7 mg/(h·k€).^24,25^ Much better ISP values have recently been achieved with a lower investment,^31,32^ but the high-power laser setups remained very expensive, which is an issue both at the laboratory level as well as for scaling up production to larger quantities. Overall, the LAL synthesis of Au NPs remained economically less advantageous than aqueous-phase chemical synthesis with the Turkevich–Frens approach and its variants.^33,34^ An economic breakeven point at an MP of 0.55 g/h has been predicted for LAL versus chemical synthesis when centrifugation is included in the latter to remove excess reactants from the colloidal solution to avoid postsynthesis NP growth.^33^ On the other hand, LAL does not allow the production of NPs with a predetermined average size, and often, the polydispersity index (PDI) is above 0.25,^15,34^ despite monodisperse metallic NPs being routinely produced using the appropriate saline environment.^15,35^

To overcome such limitations, a new perspective is offered by artificial intelligence, nowadays pervasive in science and technology, including the optimization of synthesis processes where empirical experimentation or theoretical modeling has been unsuccessful in delivering sufficient innovation.^36–38^ Machine learning (ML) is playing a significant role in catalysis, for instance, drastically reducing the computational effort of quantum mechanical models alone and experimental validation efforts, as shown with the identification of Au NPs’ active sites in the CO_2_ reduction reaction.^39^ Regarding the synthesis of metal nanoparticles, ML has been embedded in autonomous workflows for the classical Turkevich–Frens Au NP synthesis, integrating in situ X-ray spectroscopy with Gaussian-process models and Bayesian optimization, allowing closed-loop nanoparticle synthesis and fine-tuning of crystallite domain size and particle diameter.^40^ Often, small data sets are available in chemistry, a problem which has been tackled with a Random Forest ML model and Bayesian optimization to predict complex shapes (cubes, bipyramids, etc.) of Cu nanocrystals from 29 reaction parameters.^41^ Despite several examples existing on the application of ML in the chemical synthesis of NPs, only recently has it entered the field of LAL, enabling the synthesis of Cu nanostructures with a predetermined composition.^42^ Therefore, many prospects exist for the combination of ML with ongoing innovations in industrial laser technology, particularly those offering high output tunability and low laser equipment cost, to improve the competitiveness of Au NP synthesis via LAL while preserving its advantages in terms of sustainability and purity.^42–45^

Here, the productivity of the LAL synthesis for 5 nm Au NPs in water was optimized with the assistance of ML, starting from a setup made of a low-cost commercial fiber laser with broad tunability in terms of pulse duration, energy, and repetition rate. The process was implemented in a batch configuration, well-suited to the research laboratory context and gram-scale synthesis, while at the same time allowing straightforward scale-up to larger volumes. The final procedure surpassed previously reported ISPs by 3.4-fold, while also beating batch Turkevich–Frens synthesis in terms of gram-scale production costs and environmental sustainability. Most importantly, the LAL-generated Au NPs are synthesized without chemical reagents and present intrinsically clean, ligand-free surfaces. Relative to a commercial benchmark, they consistently outperform multiple orthogonal readouts, showing enhanced cytocompatibility, superior MALDI substrate performance, higher catalytic activity for nitrothiophenol reduction, increased surface coverage by thiolated molecules, and stronger plasmonic extinction. This study highlights the positive prospects of ML-optimized LAL for the low-cost and environmentally sustainable production of metal NPs possessing convenient properties that are not achievable through wet-chemistry routes.

## Results

### ML-Guided Maximization of Productivity

For the laser synthesis of Au NPs, a batch configuration was employed (Figure 1A), in which a bulk gold target was immersed in a Milli-Q water solution containing 2 × 10^–4^ M NaCl inside a glass container, and the solution was subjected to magnetic stirring throughout the process. The addition of NaCl during the LAL synthesis of Au NPs enhances their stability and promotes monodispersity.^46^ The laser beam, focused onto the target, was generated by an all-fiber random nanosecond fiber laser-seeded master oscillator power-amplifier operating in the near-infrared (1064 nm), with adjustable pulse duration (10–250 ns), repetition rate (10–250 kHz), and output power (0.67–57.1 W). The maximum output power depends nonlinearly on the pulse duration and repetition rate. An additional parameter is the scanning speed at which the laser beam is moved across the target surface by a galvanometric optical scanner (up to 8000 mm/s). This speed must exceed a minimum threshold to prevent overlap with the cavitation bubble formed at the ablation site, thereby avoiding attenuation effects that would otherwise reduce the synthesis efficiency.^22,23,25^ To identify the productivity hypersurface of Au NPs as a function of the synthesis parameters, the LAL yield was initially mapped empirically over 96 parameter combinations distributed within the ranges allowed by the experimental setup (Table S1 in the Supporting Information, S.I.). At this stage, taking advantage of the versatility of the batch configuration, both the synthesis duration and the solution volume were reduced (1–3 min, 40 mL) to accelerate the empirical mapping of the 96 experimental conditions. The Au NP concentration was quantified spectrophotometrically according to well-established procedures in the literature, validated with ICP-MS.^47,48^

**Figure 1:**
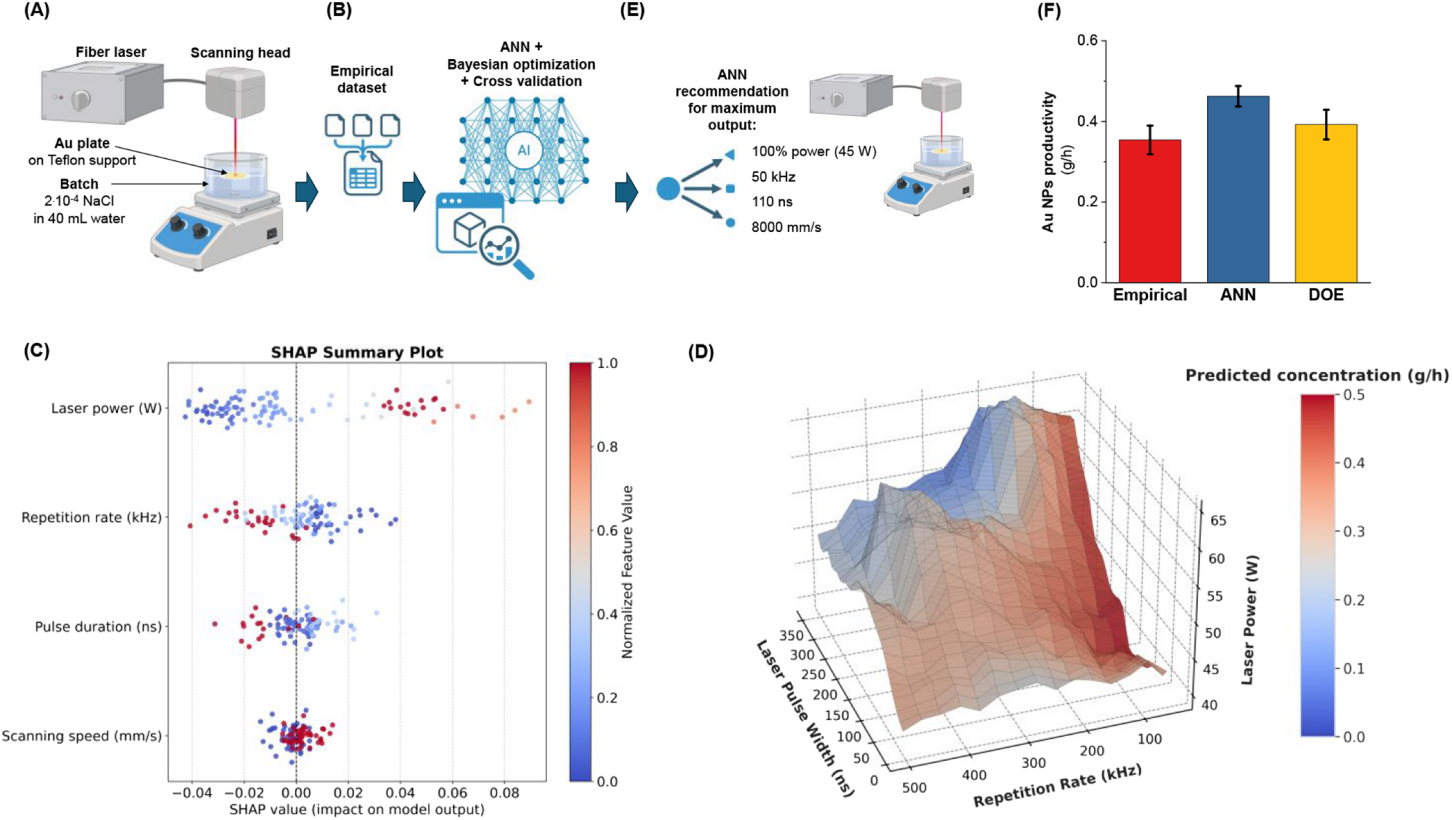
(A) Sketch of the LAL batch configuration for synthesis of Au NPs of the initial data set. (B) The experimental data set used to train an ANN. Bayesian optimization and cross-validation were applied to optimize the ANN model. (C) Feature ranking of the ML model and the related SHAP plot. (D) Au NPs’ productivity hypersurface obtained from the ML model, shown as a 3D plot illustrating the effects of laser pulse width (*x*-axis, ns), repetition rate (*y*-axis, kHz), and laser power (*z*-axis, W) for 100% laser power and at the highest allowed scan rate of 8 m/s. The surface is color-coded, indicating higher (red) and lower (blue) productivity. A 360° rotation of the 3D plot is provided in Movie S1 in S.I. Sketch (E) and histogram (F) of the experimental test of Au NPs’ productivity according to ML (and DOE) predictions. Figures A, B, and E were created with biorender.com.

The analysis of productivity as a function of the different synthesis parameters reveals a prevailing dependence on laser power, as productivity increases in all cases where the power is maximized, while the other parameters are kept constant (Figure S1 in S.I.). However, the resulting picture is considerably more complex than a trivial maximization of power since both the laser pulse duration and the repetition rate also play a key role, with trends that would not have been predictable a priori via brute force trial and error empirical investigation. In particular, the best performance is obtained for a pulse duration of 100 ns and a repetition rate of 60 kHz. Under these conditions, the scan rate appears to have a limited influence, in contrast to what is observed for shorter and longer pulse durations.

To navigate this particularly intricate parameter space and to identify a reliable model capable of guiding the selection of parameter combinations associated with maximum productivity, the experimental results were used to train an artificial neural network (ANN, Figure 1B). To enhance the model’s performance, Bayesian optimization coupled with cross-validation was employed for hyperparameter tuning, leveraging its ability to optimize ANN parameters by rapidly converging toward the optimal configuration based on R^2^ metric variations. The ANN emerged as the most accurate machine-learning model for the available experimental data set (coefficient of determination, R^2^ = 0.99 on the training set and 0.92 on the testing set). Tree-based alternatives, such as XGBoost, were tested and excluded because the predictions often tend to plateau within the sparse experimentally sampled range.^49,50^ Feature ranking of the ML model (Figure 1C) confirms the predominance of laser power over the other parameters, followed by repetition rate, pulse duration, and, to a lesser extent, scanning speed. An additional advantage of ML models is the possibility of exploiting Shapley additive explanations (SHAP) to extract physically meaningful insights into the role of the different parameters on productivity, even in a complex scenario.^36,50^ The SHAP plot (Figure 1C) clearly shows that laser power is positively correlated with Au NP productivity, whereas the repetition rate is anticorrelated. Pulse duration provides a positive contribution to productivity at intermediate values, while the scanning speed is also positively correlated. Although more complex mutual interactions between pairs of synthesis parameters can be extracted from the SHAP analysis (Figure S2 in S.I.), this is deferred to the Discussion section. At this stage, a predictor was constructed from the trained machine-learning model by ML-driven data augmentation.^38,51^ The ML model was used for mapping an extended matrix of possible synthesis-parameter combinations (Figure 1D and Movie S1 in S.I.) for 100% laser power and at the highest allowed scan rate of 8 m/s. As highlighted in the red zones of the 3D parameter space, the highest NP yields are achieved under 100–200 ns of laser pulse duration, 50–100 kHz of repetition rates, and between 40 and 50 W of laser power. From this productivity hypersurface, the most favorable region for Au NP synthesis is found at 110 ns, 50 kHz, 100% laser power (55 W), and 8000 mm s^–1^, corresponding to an estimated theoretical output of 0.48 g·h^–1^. The prediction was experimentally validated using the same setup employed for generating the training data set, showing a 30% improvement (0.46 ± 0.02 g·h^–1^) with respect to the best result obtained empirically (0.35 ± 0.04 g·h^–1^, Figure 1E-F).

This type of experimental problem is also suited for a design of experiments (DOE) approach (see Methods and Figure S3 in the S.I.). However, the DOE-based prediction yielded only an 11% improvement (0.39 ± 0.04 g·h^–1^) over the empirical result (Figure 1F and Table S2 in S.I.). Moreover, DOE does not provide easily interpretable physical insights into the role of individual parameters, as is possible with SHAP analysis.

After the conditions of maximum productivity were identified with the available setup, Au NP synthesis was carried out in a larger-volume batch (1.6 L), representative of a typical laboratory-scale or spin-off level synthesis (Figure 2A). The resulting mass productivity (MP = 0.445 ± 0.005 g·h^–1^, Figure 2B) confirms the trends observed in the training experiments. MP represents an absolute productivity metric for the specific setup considered and is therefore 8.5 times lower than the record reported in the literature for lasers with power and cost, respectively, 9- and 50-fold higher.^24,25^ The laser power-specific productivity (PSP, i.e., normalized to laser power,^22,27^ Figure 2C) is 9.84 ± 0.11 mg·h^–1^·W^–1^, exceeding 7.6 mg·h^–1^·W^–1^ reported for the 500 W setup.^24,25^ However, the highest PSP for LAL Au NPs reported in the literature are 9.5 mg·h^–1^·W^–1^, obtained using a diffractive-optics system coupled to a laser with lower power and cost (98.9 W and 203.7 k€, respectively),^31^ 21 mg·h^–1^·W^–1^, obtained using the 500 W laser setup with the polygon scanner but operated at 60 W,^24^ and 18 mg·h^–1^·W^–1^, obtained with a less expensive ns laser equipped with a galvanometric scanner (242 ± 60 k€; see Figure S4 for details) operated at 121 W.^32^ Notably, the productivity normalized to the investment required for the synthesis setup is significantly higher than literature benchmarks: here, ISP reaches 33.0 ± 0.4 mg·h^–1^·k€^–1^, surpassing the costly state-of-the-art system by a factor of 6, the more recent and lower-cost diffractive-optics-based system by a factor of 7, and the record ISP value achieved with the ns laser and galvanometric scanner by a factor of 3.4 (Figure 2D).

**Figure 2:**
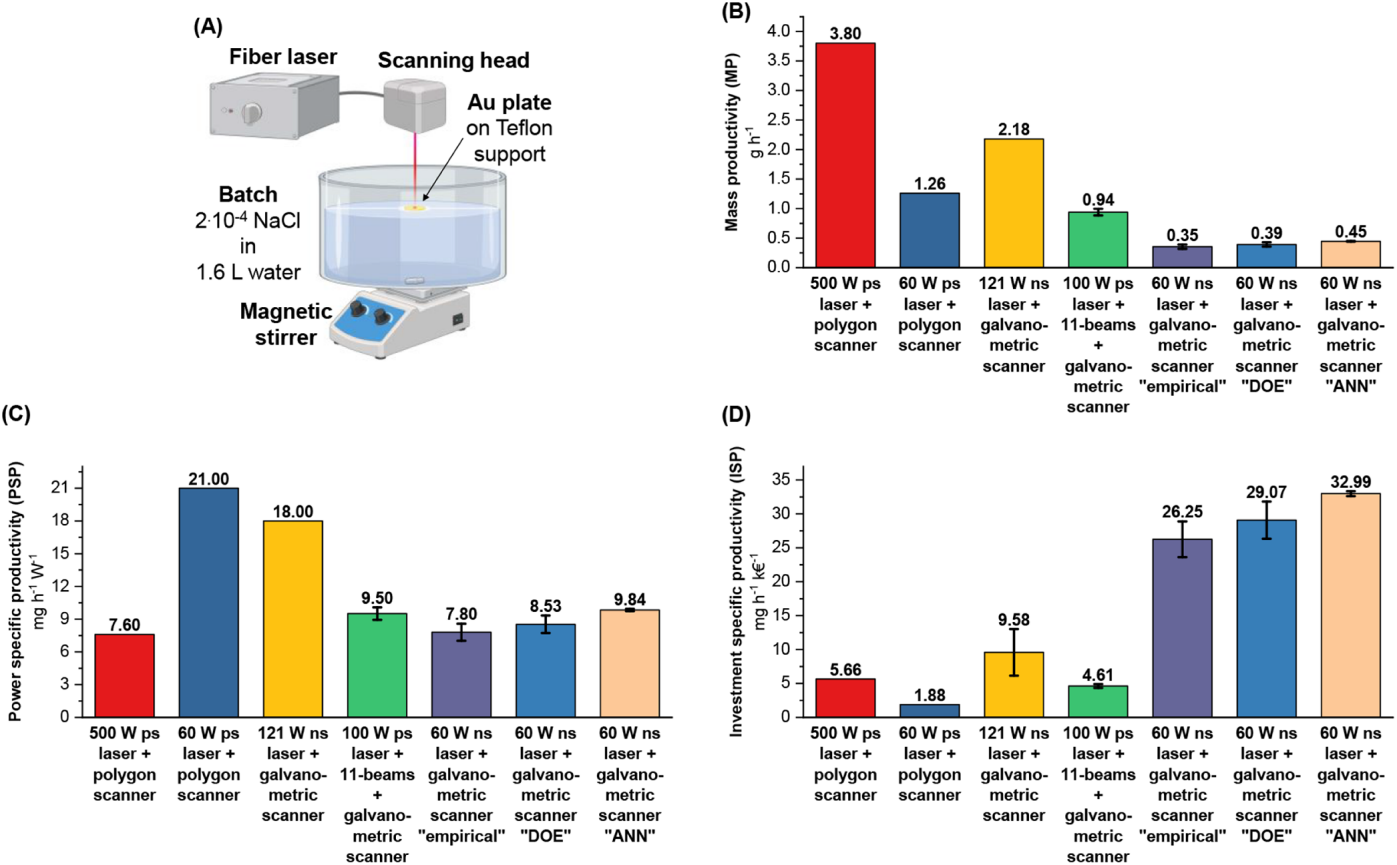
(A) Sketch of the LAL synthesis of Au NPs in a 1.6 L batch using the parameters optimized with the ML model. Created with biorender.com. Comparison of mass productivity (B), power-specific productivity (C), and investment-specific productivity (D) for this study and other benchmark LAL Au NP synthesis systems.

### Comparison with Au NPs from Turkevich–Frens Synthesis

The UV–visible absorption spectrum of the Au NPs obtained by LAL in the large-batch configuration is characterized by a localized surface plasmon resonance (LSPR) band at 519 nm, typical of spherical particles (Figure 3A). Since the Au NPs are produced in Milli-Q water containing only 10^–4^ M NaCl, without any additional additives or chemical compounds, the spectral region associated with Au interband transitions is also clearly observable down to 200 nm without interference. This represents a distinctive feature of LAL-derived Au NPs and is not compatible with nanoparticles obtained by chemical reduction methods.^10,14,33,47^

**Figure 3:**
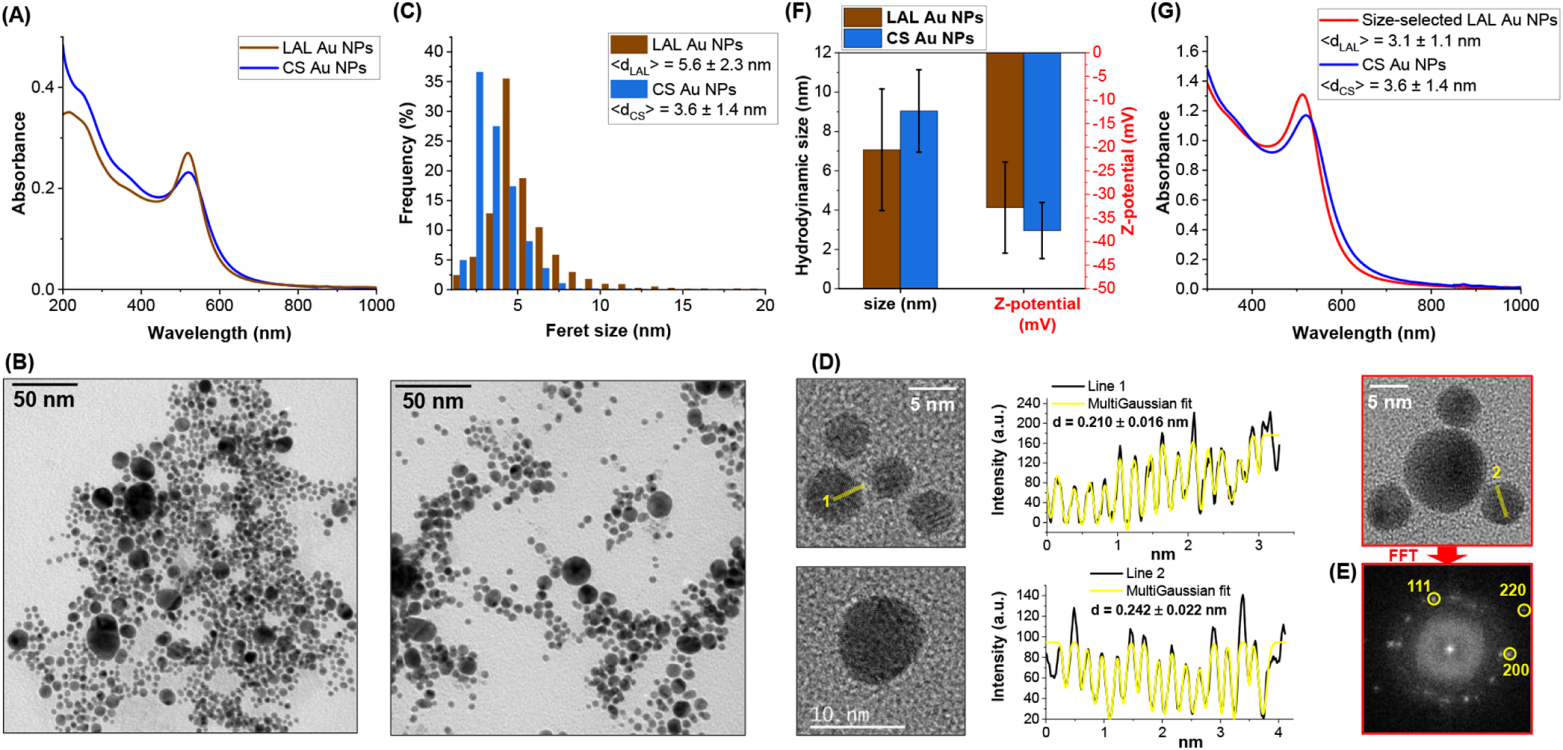
UV–vis absorption spectrum (A), representative TEM image (B), and size histogram (C, *N* > 1500) of the LAL Au NPs. In addition, Figures (A) and (C) report, respectively, the UV–vis and size histogram (*N* > 1500) of a commercial CS Au NP sample (nominal size 5 nm; see Figure S6A in S.I. for a representative TEM image of CS Au NPs and Methods for product details). HRTEM (D) and associated FFT of the regions in the red squares (E) of LAL Au NPs. (F) Average hydrodynamic size and surface Z-potential of LAL and CS Au NPs. (G) UV–vis spectra, normalized at 400 nm for easier comparison, of the size-selected LAL (3.1 ± 1.1 nm, PDI = 0.126) and commercial CS Au NPs (3.6 ± 1.4 nm, PDI = 0.151), showing a more intense LSPR for the LAL sample.

Transmission electron microscopy (TEM, Figure 3B) confirms the spherical morphology of the Au NPs, indicating an average particle size of 5.6 ± 2.3 nm (PDI = 0.169). The size distribution measured by TEM (Figure 3C) exhibits the typical log-normal shape associated with NP formation via LAL, also including a small fraction of larger particles arising from the ejection of molten metal droplets from the target.^52–54^ The small size of the Au NPs is a general outcome of the employed setup and is only weakly affected by the synthesis parameters, as evidenced by the violin plot of the mean Au NP sizes across the training data set (Figure S5 in the S. I.), which shows a median size of 5.0 nm with a maximum spread of ± 1.3 nm.

The Au NPs exhibit a face-centered cubic (fcc) structure and are clearly polycrystalline, with several grain boundaries among the individual monocrystalline domains within each NP (Figure 3D). The interplanar spacings characteristic of gold are identified by the line profiles 1 and 2 in Figure 3D, corresponding to, respectively, the (200) and (111) interplanar spacings of fcc Au. This is further confirmed by the associated FFT pattern from the region delimited by the red square in the HRTEM images (Figure 3E), identifying the (111), (200), and (220) reflections of fcc Au. According to molecular dynamics simulations, the polycrystalline structure arises from the rapid cooling of the nanoparticles following material ablation from the target, as well as from the coalescence of fragments ejected from the target through phase explosion and photomechanical mechanisms.^52–55^

In Figure 3C, for comparison, the size distribution of a commercial citrate-stabilized (CS) Au NP sample (nominal size of 5 nm) is shown. TEM analysis of the CS-Au NPs indicates an actual particle size of 3.6 ± 1.4 nm (PDI = 0.151) with a log-normal size distribution, confirming that the Turkevich–Frens method produces samples with polydispersity that, at least in this small-size range, is slightly lower than LAL Au NPs. DLS and Z-spectroscopy analyses (Figure 3F) also demonstrated comparable hydrodynamic size and surface Z-potential for the LAL (7.1 ± 2.1 nm, −33 ± 10 mV) and CS Au NPs (9.0 ± 2.1 nm, −38 ± 6 mV). The hydrodynamic size of CS Au NPs is larger than the nominal size of 5 nm, the Feret size of 3.6 nm, and the hydrodynamic size of LAL NPs, and it may be attributed to the formation of small clusters of NPs. While the negative charge of CS Au NPs is due to adsorbed citrate ions, that of LAL Au NPs is due to adsorbed Cl^–^ ions from the NaCl dissolved in solution for this specific purpose and adsorbed OH^–^ groups generated from water decomposition during laser ablation.^9,15,35,56^

A notable feature emerges when the absorption spectra of the CS-Au NPs are compared with the LAL Au NPs (Figure 3A), as the plasmon resonance of the citrate-stabilized sample is significantly weaker. To determine whether this lower LSPR originates from the differences in the size distribution or is due to the different synthesis route, the LAL sample was subjected to size selection via selective sedimentation for isolating a fraction with a size distribution comparable to that of the commercial sample (Figure S6 in the S.I.). This procedure has been previously conceived to sort chemical-free NPs without any additional solute or stabilizer.^57^ The spectrum of this fraction of LAL Au NPs, with an average size of 3.1 ± 1.1 nm (PDI = 0.126, lower than the 0.151 of the CS NPs), still exhibits a markedly stronger LSPR (Figure 3G), confirming that this difference arises from the surface chemical composition of the Au NPs. In CS Au NPs, although only trace amounts of Au^δ+^ species have been experimentally detected at the particle surface,^58^ the negative charge of the carboxyl groups introduces polarization effects in the metal.^59^ Furthermore, XANES measurements have revealed changes in the surface electronic states of the CS-Au NPs, indicative of structural disorder, shorter bond distances, and the presence of unoccupied d-states.^60^ These factors are known to contribute to chemical interface damping of the LSPR.^6,61,62^ The effects of the adsorbed citrate ions are not present in the LAL-derived Au NPs, which therefore retain superior plasmonic properties, crucial for prospective photothermal and photocatalytic applications.^63,64^ As a further control, citrate buffer was added to the LAL-derived Au NPs, leading to a moderate increase in the plasmon resonance and a slight broadening of the absorption band (Figure S6D). This test indicates that the chemical interaction of citrate ions with the LAL Au NP surface is not equivalent to that of citrate ions adsorbed on CS Au NPs during the reduction of gold ions.

The differences in surface chemistry can lead to further significant properties and performance in typical applications of Au NPs, particularly in functionalization with thiolate molecules. This aspect was tested using mercaptoundecanoic acid (MUA), a thiol commonly employed in the literature to probe surface functionalization of Au NPs through the Au–S chemical bond.^65,66^ UV–vis analysis of the two types of Au NPs (LAL and CS, Figure 4A) indicates that LAL Au NPs exhibit a larger red-shift of the LSPR following the addition of the thiolate ligand (5.0 ± 0.5 nm versus 0.5 ± 0.5 nm for CS NPs). This red-shift is primarily attributed to the increase in surface permittivity of the Au NPs and may also reflect chemical interface damping due to the formation of Au–S bonds.^6,65,66^ The surface functionalization of Au NPs with MUA was further confirmed by X-ray photoelectron spectroscopy (XPS, Figure 4B,C), showing an S/Au ratio of 0.45 for LAL Au NPs compared to 0.26 for CS Au NPs. This result indicates a higher reactivity of LAL Au NPs toward thiolate molecules than CS NPs, likely due to the persistent adsorption of citrate molecules on the surface of the latter. XPS data support this hypothesis, as the S:Na ratio is 0.12 for CS Au NPs versus 0.25 for LAL Au NPs. The presence of Na^+^ near the surface of the CS Au NPs, acting as counterions to citrate ions adsorbed on the overall neutral gold surface, has been experimentally observed and computationally validated.^58,59^ Surveys also confirm that CS Au NPs have more O and C compared to LAL Au NPs, which is compatible with the presence of adsorbed citrate ions (Figure S7 in S.I.).

**Figure 4:**
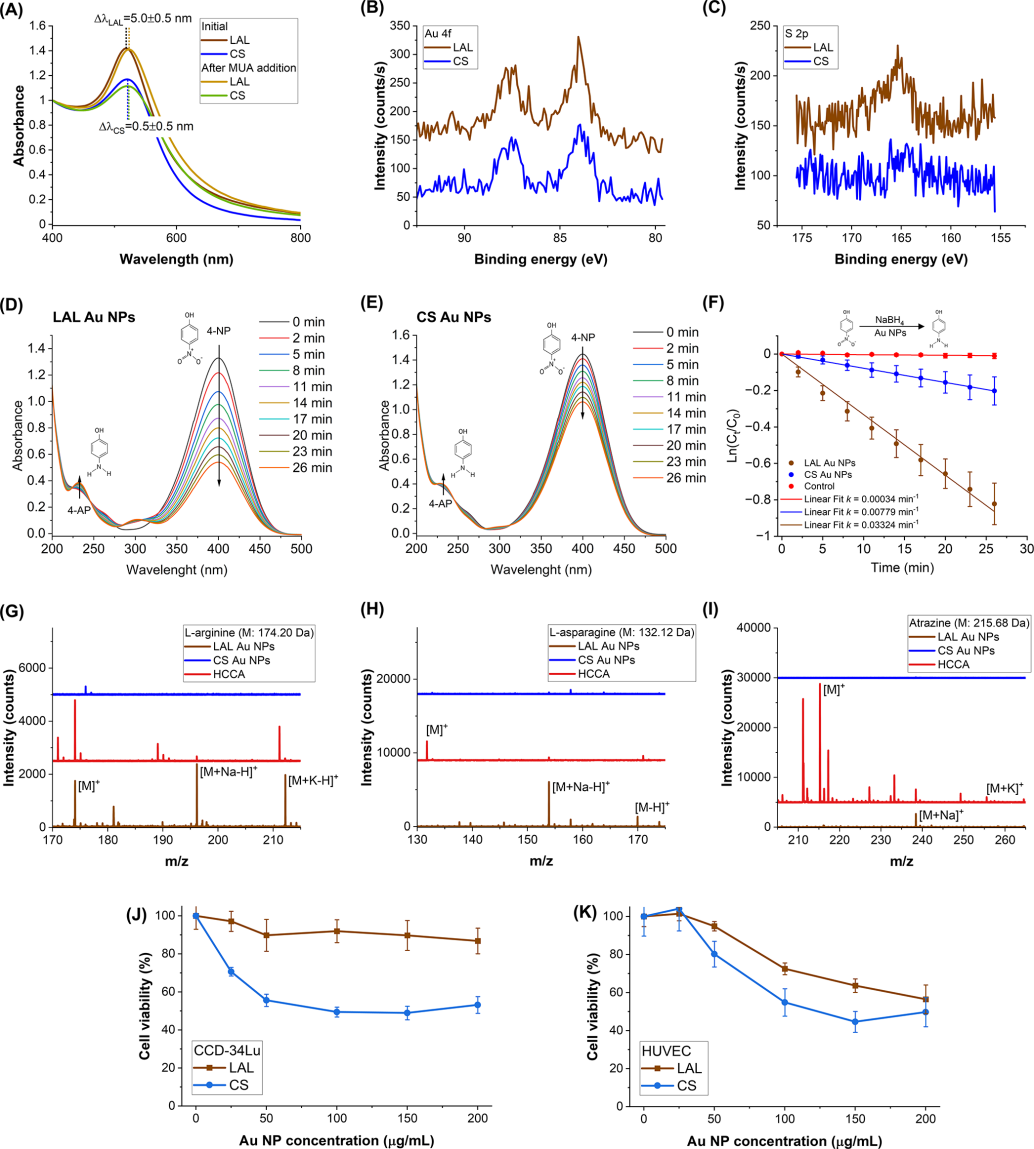
(A) UV–vis absorption spectra of LAL and CS Au NPs after coating with MUA. Spectra were normalized at 400 nm for easier comparison. XPS analysis of S 2p (B) and Au 4f (C) peaks in LAL (black) and CS (blue) Au NP samples. Catalytic reduction of 4-NP with NaBH_4_ by LAL (D) and CS (E) Au NPs is indicated by the gradual reduction of the 4-NP band at 400 nm, while that of 4-AP at 230 nm increases. (F) The comparison of the pseudo-first-order kinetic model fitting of all samples shows the superior performance of the LAL Au NPs. MALDI spectra of three low-mass analytes, l-arginine (MW 174.20, G), l-asparagine (MW 132.12, H), and atrazine (MW 215.68, I), were recorded using three different matrices: LAL Au NPs (black), CS Au NPs (blue), and HCCA (green). The cytotoxicity of LAL and CS Au NPs was assessed by using the MTS assay in normal lung fibroblasts (CCD-34Lu, J) and human endothelial cells (HUVEC, K) for an incubation period of 48 h.

The availability of small, clean Au NPs, free of additives or stabilizers, represents an ideal condition for catalytic applications of any type.^13,18,63,64^ Therefore, the catalytic activity of the two types of Au NPs was evaluated by using the model catalytic reduction of 4-nitrophenol (4-NP) to 4-aminophenol (4-AP) in the presence of sodium borohydride (NaBH_4_). This reaction follows pseudo-first-order kinetics under conditions of excess NaBH_4_,^13,67–69^ facilitating the determination of the activation energy and enabling a straightforward assessment of the overall catalytic behavior.^13,69^ To perform this experiment under well-reproducible conditions, the Au NPs were quantitatively pelleted via centrifugation after mixing with a polymer scaffold based on SH-functionalized dextran.^70,71^ This procedure removed sodium citrate and other additives present in the solution of the commercial CS Au NP, while keeping the same pellet preparation conditions as those for the LAL sample. The results indicate significantly higher catalytic activity for LAL Au NPs (Figure 4D,E), with the consistent reduction of 4-NP absorption at 400 nm, while 4-AP increases at 230 nm. In the LAL Au NP sample, a rate constant of 3.32 ± 0.07 × 10^–2^ min^–1^ was measured (Figure 4F), compared to 7.79 ± 0.06 × 10^–3^ min^–1^ for the CS sample, which still shows appreciable activity relative to the control (rate constant 3.4 ± 0.8 × 10^–4^ min^–1^, Figure S8). It is worth noticing that, in several cases, laser-generated colloids have higher defect densities than chemically synthesized equivalents, and this structural feature was associated with higher catalytic activity.^15,18,34^ LAL Au NPs also have several grain boundaries (Figure 3), which are expected to contribute to their catalytic activity. However, CS Au NPs have a smaller average size (3.6 ± 1.4 nm) and a larger surface-to-volume ratio than the LAL Au NPs (5.6 ± 2.3 nm). This factor was expected to lead to a more consistent catalytic activity for the CS sample in the absence of the adsorbed contaminants on the particle surface originating from the different synthesis methods. The hydrophilicity of the LAL Au NP surface, also due to the presence of adsorbed OH^–^ groups,^9,15,35,56^ may further facilitate the adsorption of polar molecules such as 4-NP,^13,67–69^ contributing to the observed catalytic performance.

The superior purity of LAL Au NPs is of particular interest in biomedical and analytical applications, where even simple contaminants such as sodium citrate can produce undesired effects.^2,11,12^ For example, metallic NPs are known to serve as matrices for analyte detection via matrix-assisted laser desorption/ionization (MALDI).^14,72,73^ A significant limitation of MALDI is the detection of low-mass analytes (<500 Da) due to interference from fragments arising from the standard organic matrices.^14,73^ LAL and CS Au NPs were tested for this application using low-mass analytes, including the amino acids l-arginine (MW 174.20) and l-asparagine (MW 132.12) and the pesticide atrazine (MW 215.68). The results (Figure 4G–I) demonstrate the good performance of LAL Au NPs owing to the negligible presence of matrix-related peaks and high signal intensity. In contrast, CS Au NPs produced weak signals, insufficient for use as a MALDI matrix, even though dialysis membranes were used to reach the optimal concentration range reported in the literature^14^ without increasing the molarity of additives present in the commercial product. As a reference organic matrix, α-cyano-4-hydroxycinnamic acid (HCCA) was used because it is a standard in MALDI mass spectrometry and commonly applied for low-molecular-weight peptides and small molecules. Nevertheless, HCCA produced signals either weaker than those of the LAL Au NPs or accompanied by several matrix-derived peaks. This is an undesirable condition for unambiguous analyte identification,^14,72,73^ which was effectively absent in the spectra of the LAL Au NPs.

Finally, the cytocompatibility of the two types of Au NPs was evaluated by using normal pulmonary fibroblasts (CCD34-Lu) and primary human umbilical vein endothelial cells (HUVECs), incubated for 48 h at concentrations up to 200 μg·mL^–1^ (Figure 4J,K). As for the MALDI experiments, to reach the required starting concentration of the colloid (2 mg·mL^–1^), the CS Au NPs were concentrated via dialysis to avoid increasing the stabilizer content relative to that of the original commercial product. Instead, LAL Au NPs were concentrated to the same level using a standard DNA concentrator, without producing waste of consumables for dialysis (see Methods) and with negligible labor required. Despite the precaution on CS NPs’ concentration, LAL Au NPs exhibited higher cytocompatibility than CS Au NPs in both cell lines. This result is particularly relevant, given that CS Au NPs are generally considered nontoxic,^2,74,75^ highlighting once again the benefits of the absence of chemical additives in solution or adsorbed on the surface in LAL Au NPs. In the case of CS Au NPs, the gold surface contains residual amounts of tetrachloroaurate, chloride, and hydroxyl anions, as well as byproducts from citrate oxidation (dicarboxyacetone and other smaller intermediates),^76^ which have been associated with negative effects on cell health.^33,75,77^ In addition, LAL Au NPs have a hydrophilic surface and undergo rapid coverage with serum proteins such as bovine serum albumin, which enhances their biocompatibility.^9,15^

## Discussion

### LAL Mechanistic Aspects

The limited scalability of LAL remains the primary barrier to its widespread adoption in nanomaterials production. Here, the highest ISP ever reported for the LAL of Au NPs has been achieved using ML guidance and starting from a low-cost setup based on a fiber laser emitting 1064 nm pulses in the nanosecond regime, with adjustable pulse duration, repetition rate, and power, coupled to a standard galvanometric optical system and employing a simple batch configuration. Even a DOE approach (Figure S3 in S.I.) proved less versatile than the ANN model. This underscores the importance of using ML, which not only maximized productivity by navigating the complex synthesis-parameter hypersurface but also provided a direct interpretation of the role of different experimental parameters through SHAP analysis. Beyond the expected positive correlation between productivity and laser power, SHAP highlights the less intuitive anticorrelation with repetition rate and identifies a “sweet spot” for pulse duration around 100 ns, an intermediate value within the experimental setup allowed range. The complex correlations between pairs of synthesis parameters shown in the SHAP plots (Figure S2 in S.I.) are the result of the interconnected effects of the output power and pulse energy intrinsic to the fiber laser and of the consequent mechanistic effects related to laser pulse energy and laser shielding phenomena. The productivity trends versus individual parameters or their pairs are insufficient to elucidate such complex relationships or to empirically guide the operator toward maximum output, unless an extensive, time- and resource-intensive trial and error experimental effort is undertaken. For instance, in the experimental data set for training the ML model, the highest productivity is not achieved at maximum laser power (Figure S1 in S.I.) or energy per laser pulse (Figure S9 in S.I.). In addition, when looking at the condition of maximum productivity for each pulse duration in the data set, it is found that laser pulse energy peaks above 100 ns (Figure S10A in S.I.), and these peaks are found at low repetition rates (Figure S10B in S.I.), whereas the pulse energy is uniform against repetition rate for shorter pulses (10, 20, 30 ns), with only a moderate increment at 60 ns. These trends, intrinsic to the fiber laser output (Figure 5A), interpenetrate with the mechanistic factors involved in the LAL process, which critically depend on the pulse duration, repetition rate, and scanning speed, overall determining the fraction of energy delivered to the target versus that lost due to thermal dissipation or laser beam shielding. The first of these factors is that, for pulse duration longer than the electron–phonon relaxation time of gold (∼ 1 ps), the increase in pulse duration is accompanied by a decrease in the ablation efficiency because part of the energy is dissipated to the metal lattice (Figure 5B).^22,27^ The thermal penetration depth is above 1 μm for pulse durations above 10 ns, reaching 5 μm at 110 ns and exceeding 8 μm at 250 ns (Figure S11 in the S.I.), indicating significant thermal losses for the longest pulse duration. The second factor is plasma shielding by the plasma plume, which arises a few nanoseconds after the beginning of the laser-matter interaction (Figure 5C).^23,78–80^ The plasma plume can absorb or scatter the final part of the incoming laser beam, lowering ablation efficiency.^22,23,54^ Therefore, plasma shielding is particularly relevant for pulses with durations of several hundred nanoseconds.^23,78–80^ The third factor is cavitation bubble formation on time scales exceeding hundreds of nanoseconds, attenuating the intensity of the subsequent laser pulse via light scattering.^54,81,82^ The cavitation bubble is due to heating and vaporization of the liquid near the target, creating bubbles with size and lifetime influenced by the absorbed laser energy^54,83^ and the physical and chemical properties of the liquid.^84,85^ The cavitation bubble can be bypassed only when the scanning speed is large enough compared to the laser repetition rate (Figure 5D).^22,25,28^ In the ANN-optimized conditions (50 kHz and 8000 mm/s), the interpulse distance is 160 μm, becoming only 32 μm at 250 kHz. The fourth factor is due to particle clouds and persistent microbubbles formed during ablation (Figure 5E) which can accumulate near the target, creating a dense layer that scatters or absorbs laser light, further reducing penetration to the target.^15,22,29^ This factor is more relevant at high laser pulse energies and low repetition rates.

**Figure 5:**
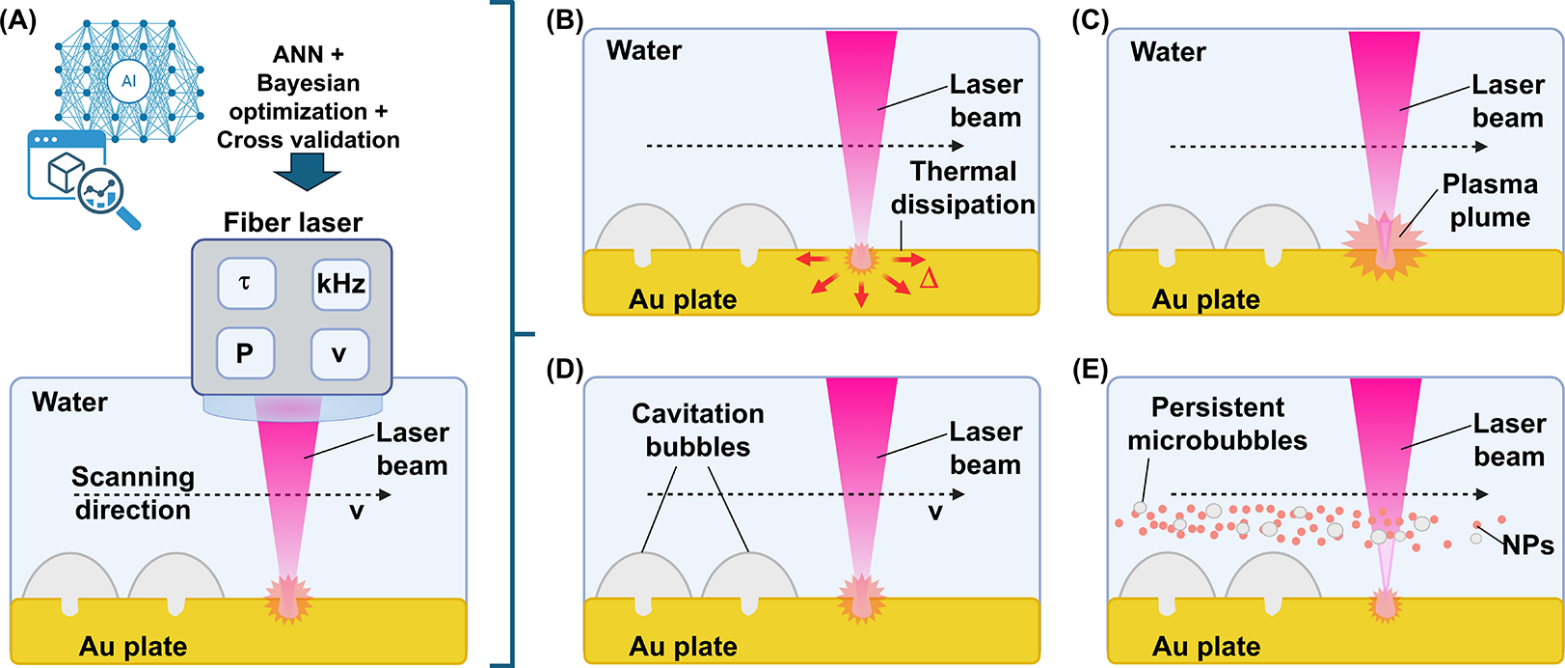
Sketch of the five aspects to be considered for optimization of LAL productivity with the setup of this study: (A) dependence of laser power (P) on pulse duration (τ) and repetition rate (kHz), which have been optimized by the ANN together with the scanning speed (v), accounting also for the next phenomena; (B) thermal penetration depth, determining energy dissipation as heat (Δ) for pulse durations much longer than the electron–phonon relaxation time of gold (∼1 ps); (C) plasma shielding, i.e., the attenuation of laser pulse energy due to the plasma plume generated since the first tens of ns after laser-target interaction; (D) cavitation bubble bypass, required at high repetition rates to avoid scattering of laser pulses from cavitation bubbles generated by previous laser pulses; (E) scattering and attenuation of laser pulses due to NPs and persistent microbubbles generated by previous laser pulses. Created with biorender.com.

To obtain experimental insight into the LAL in the different conditions of the experimental data set, single-pulse laser-ablation crater analysis was performed on a flat Au plate in the same conditions as the LAL synthesis (Figure S12 in S.I.). With reference to the experimental data set, the analysis was performed in the conditions of the highest output power for 10, 20, 30, and 60 ns pulse durations (Figure S12A in S.I.) and with 100 and 250 ns pulses after setting their single-pulse energy at a similar value of the 20, 30, and 60 ns series (Figure S12B in S.I.). The experiment was also performed with 100 and 250 ns pulses under the conditions of highest productivity (Figure S12C in S.I.). Finally, the ANN conditions with 110 ns pulses were also tested in the optimized LAL configuration and at single-pulse energy equivalent to the 20, 30, and 60 ns series (Figure S12B–C in S.I.). The series at comparable pulse energy shows that crater diameter and depth are larger for a pulse duration of 60 ns than for 100–250 ns (Figure S12D in S.I.), which was expected considering the increase of thermal penetration depth with pulse duration. However, for 10, 20, 30, and 60 ns, the laser spots are close and partially overlapping (Figure S12A in S.I.) because the highest power is achieved at a high repetition rate. This condition is not favorable for productivity because of the above-mentioned light scattering and shielding phenomena. The crater dimensions are consistently larger for the 100, 110, and 250 ns pulses in the highest productivity conditions (Figure S12E in S.I.). In particular, the largest crater diameters and depths are measured at 100 ns among the empirical data set, which is the condition with the highest productivity, and at 110 ns, which is the optimal condition according to the ANN prediction. At 250 ns, the crater is smaller than at 100 ns despite the larger single-pulse energy, and a consistent portion of the material is observed on the crater edges (Figure S12A in S.I.). This is attributed to the longer pulse duration and the estimated thermal penetration depth of 8 μm (Figure S11 in the S.I.). Also, for the 250 ns pulses, there is a consistent difference between the average crater diameter measured at SEM (topographic measurement, larger) and at profilometer (depth profile, smaller, Figure S12E in S.I.).

Therefore, the ANN was efficient in learning from the experimental data set and providing the recommendation corresponding to the best compromise between high power and high pulse energy (Figure S10A in S.I.) while limiting the pulse duration and repetition rate to minimize interference from thermal dissipation and shielding phenomena. Notably, this happened without the need for the conspicuous, multitechnique, time-consuming experimental efforts required for single-pulse analysis and related interpretation in the various experimental conditions of the data set, plus all the expected iterations required for the refinement of conditions to empirical maximization of productivity. According to the single-pulse laser spot diameter measured with SEM and profilometer, fluence in the 110 ns optimized LAL conditions is between 50 ± 24 J/cm^2^ and 67 ± 18 J/cm^2^ (Figure S12F in S.I.). This range is above the threshold for phase explosion, which is evaluated at 28 J/cm^2^ for 110 ns pulses (Table S3 in S.I.).

### LAL Economic and Environmental Profile

Motivated by the large ISP, and since the experimental setup of this study meets the typical requirements of a research laboratory or spin-off for daily gram-scale synthesis, the capital expenditure (CAPEX) and operating expenditure (OPEX) were calculated to assess the initial investment and steady-state operating costs for Au NP production via LAL (Figure 6A). The analysis was carried out using the parameters reported in Table S4 in S.I., referring to the production of 2 g of Au NPs per day over 250 working days in a year (research-lab or spin-off scale), and compared with the Turkevich–Frens batch procedure for an equal daily amount of CS Au NPs with equivalent size (Figure 6B and Table S5 in S.I.). The Turkevich–Frens batch synthesis was selected because it is the conventional method for the production of Au NPs.^7,8,86,87^

**Figure 6:**
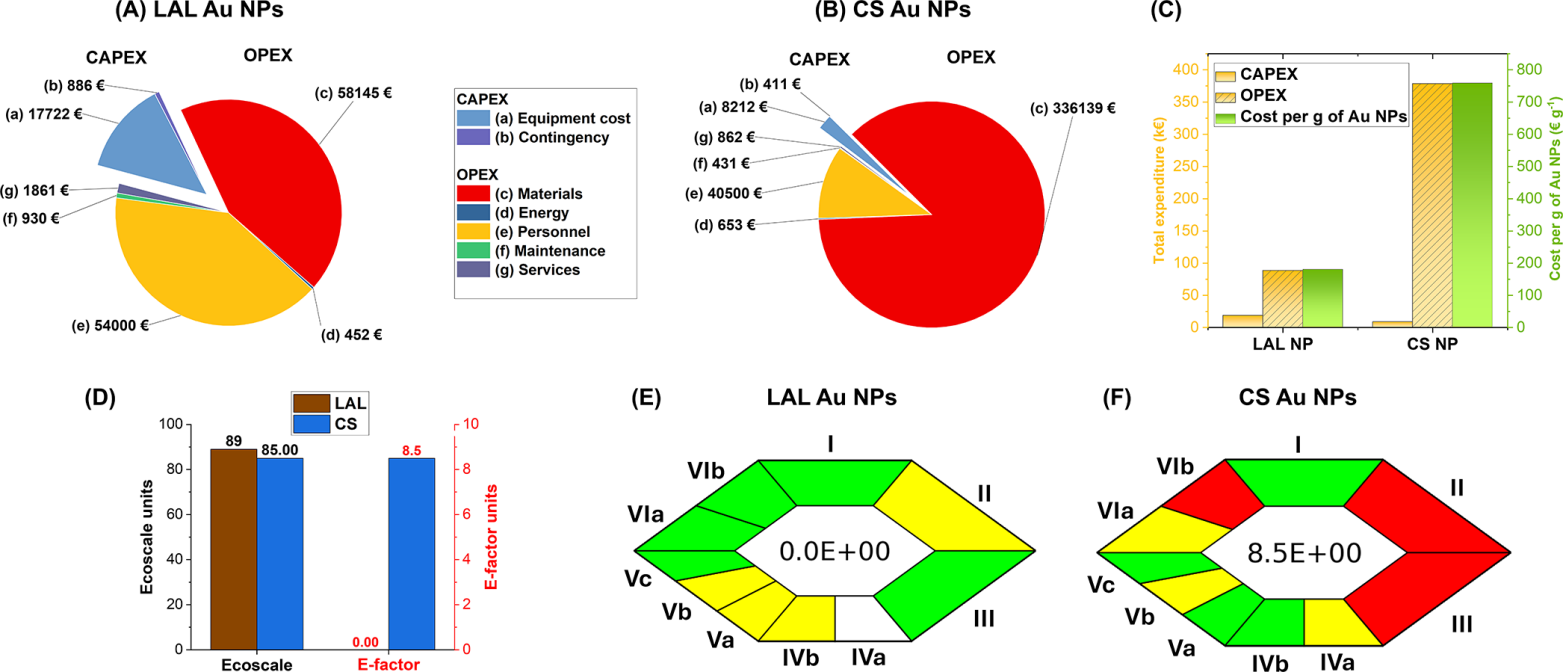
Capital (CAPEX) and operational (OPEX) expenditure pie charts for LAL (A) and CS (B) Au NP synthesis, on the assumption of a daily production of 2 g of Au NPs for 250 working days in a year (see also Tables S4-5 in S.I. for details). (C) Comparison of CAPEX, OPEX, and cost per gram of LAL and CS Au NPs. (D) Comparison of ecoscale and E-factor values for LAL and CS Au NP synthesis. The score of an ideal green process is 100 for the ecoscale and 0 for the E-factor. ComplexGAPI-based sustainability evaluation of LAL (E) and CS (F) Au NP synthesis. The pictogram adopts a four-level color code from white to green, yellow, and red to identify, respectively, null, low, medium, and high environmental impact of the process. Blocks are grouped into five categories: yield and conditions (I, II); relation to green economy (III); reagents and solvents (IVa,b); instrumentation (Va,b,c); workup and purification (VIa,b). The inset reports the E-factor value. See Table S7 in S.I. for details.

The total CAPEX includes the initial investment costs for equipment and installation (contingency 5%). Given the relatively low cost of the experimental setups in both synthesis routes, the LAL synthesis requires just 10 000€ more than the Turkevich–Frens. This CAPEX is more than 1 order of magnitude lower than benchmark high-throughput LAL systems.^24,25,31,32,88^

OPEX represents the time-based recurring costs of an NP synthesis system, including energy consumption, labor cost, maintenance cost, and consumable cost, considered over a fixed operating period of 1 year. The direct costs in the OPEX for LAL Au NPs are dominated by the bulk gold target (99%, Figure 6A). Analogously, for CS Au NPs, the gold salt precursors are the most expensive item (71%). Indeed, gold salts are more expensive than bulk gold at parity of metal mass,^33^ contributing to the high prices for commercially available Au NPs (USD 10–200 per mg).^87^ A significant contribution to direct costs also arises from chemicals used for glassware cleaning (26%, Figure 6B). It is worth noting that, for these chemicals, a conservative assumption was adopted, that is, the use of diluted stock solutions in water of aqua regia (10-fold) and isopropanol (2-fold), for a volume equal to the batch for each solution and just a single wash of the glass reactor. In the case of LAL, the OPEX considered a washing procedure after each synthesis consisting of ultrasonication with Milli-Q water (volume equivalent to five times the batch). In fact, the formation of permanent gold deposits on the walls of glassware was not observed during LAL experiments if glassware was cleaned with ultrasounds just after the synthesis due to the stability of laser-generated colloids and the synthetic route starting from bulk metal instead of nucleation and growth through chemical reduction of the metal salt.

The indirect costs in the production of OPEX are driven by personnel expenses for both LAL and CS Au NPs. However, the operations required for wet-chemistry synthesis are significantly more demanding than those required for LAL.^8,22,33^ In particular, the Turkevich–Frens method requires daily preparation of fresh reagent solutions in addition to the aforementioned accurate cleaning of glassware for each batch. These factors are critical for reproducibility and size control with the Turkevich–Frens method in general and for 5 nm NPs in particular since this is at the lower edge of the achievable sizes.^7,8,86,87^ The lower labor required for LAL Au NP production widely compensates for the higher initial setup investment already in the first production year. Nonetheless, the direct costs (89%) dominate over indirect costs (11%) for the CS Au NPs. For LAL Au NPs, indirect costs account for 34% of the OPEX, which is lower by a remarkable 76% compared to CS Au NPs, corresponding to a convenient Au NPs’ production cost of 180.3 €/g (Figure 6C).

It should be noted that waste disposal costs were not included in the final OPEX calculation. These costs are mainly associated with solutions used for cleaning glassware and laboratory tools and are difficult to quantify, as they depend on variables that are not known a priori because they are specific to each laboratory.^8^ Nevertheless, it is evident that waste disposal costs are substantially higher for CS Au NP synthesis due to the use of acids and isopropanol for the stringent glassware cleaning required to ensure synthesis reproducibility.^7,8,86,89^

It has been previously calculated that LAL becomes more convenient than chemical reduction to produce Au NPs when exceeding an MP of 0.55 g/h, if centrifugation is included in the chemical synthesis to remove excess reactants from the colloidal solution to avoid postsynthesis NP growth.^33^ The break-even MP value was lowered in this study, even without including centrifugation in the chemical reduction procedure, thanks to the guidance of ML applied to low-cost industrial fiber lasers. Considering also the affordable CAPEX, in addition to the excellent OPEX, scaling the synthesis to higher production volumes can be straightforwardly achieved by the serial replication of the LAL setup. The direct costs associated with the initial investment for LAL NP production are only 116% higher than the minimal costs required for CS Au NP synthesis and are more than compensated for by the lower OPEX already in the first year.

Sustainability is another fundamental requisite for large-scale production in modern times.^90–92^ The sustainability of the synthesis was assessed using two independent models widely adopted in the literature, namely the EcoScale^92,93^ and the Complex Green Analytical Procedure Index (ComplexGAPI).^94^ Owing to the reduced use of chemical reagents and the lower generation of waste, LAL synthesis of Au NPs achieves higher sustainability scores than the Turkevich–Frens route, which is nevertheless generally regarded as a relatively green process due to its simplicity and operation in aqueous media. The EcoScale value for LAL Au NPs is 89 versus 85 for CS Au NPs, where the score of an ideal green process is 100 (Figure 6D and Table S6 in S.I.).^92,93^ In both cases, the extraction of gold affects the environmental score,^8^ which is further lowered by the additional chemical reagents for CS Au NPs.

The ComplexGAPI chart, for the part related to the preparation of a given compound, provides a pictogram with a four-level color code from white to green, yellow, and red to identify, respectively, null, low, medium, and high environmental impact of the process. Thus, the charts (Figure 6E,F and Table S7 in S.I.) visually identify the clear sustainability advantage of LAL compared to the Turkevich–Frens batch method. The main reason is that LAL solely utilizes water with 2·10^–4^ M NaCl and bulk gold, whereas chemical reduction relies on chloroauric acid, which is classified as moderately toxic and corrosive. It should be noted that the acids and isopropanol required for glassware cleaning in chemical reduction syntheses^7,8,86,89^ were excluded from the evaluation of the green metrics for the synthesis. However, these chemicals entered the E-factor (Figure 6D),^92,94^ which is the mass of waste per mass of desired product, typically excluding water. The use of additional chemical reagents or nonaqueous solvents, as required in seeded-growth or two-phase procedures,^7,8,86,89^ would inevitably worsen the sustainability balance of chemical reduction routes based on gold salts compared to LAL.^7^ Instead, the sustainability of the LAL approach complements the intrinsic purity of the resulting Au NPs at the origin of the superior performance demonstrated in the previous section (Figure 4).

## Conclusions

Au NP synthesis procedures that are green, cost-effective, and yield NPs with clean surfaces are in high demand. Here, ML was exploited to optimize the productivity of the LAL synthesis for 5 nm Au NPs in water, thus overcoming the previous bottlenecks of this technique in terms of ISP and initial investment. The setup is made of a low-cost commercial fiber laser with broad tunability implemented in a batch configuration, well-suited to the research laboratory context and daily gram-scale synthesis, while at the same time being compatible with linear scale-up to larger production volumes. The final procedure surpassed previously reported ISPs by 3.4-fold, also beating the batch Turkevich–Frens synthesis in terms of environmental sustainability and achieving 4.2-fold lower production costs. Most importantly, the LAL-generated Au NPs are chemical-free and surface-clean, explaining the better performances in terms of cytocompatibility, analyte detection when used as MALDI substrates, catalytic activity in the reduction of 4-NP, surface thiol coverage, and a more intense plasmon absorption than equivalent commercial NPs from the Turkevich–Frens synthesis.

This achievement encourages the use of ML guidance for solving the persisting LAL challenges, also expanding the approach by including additional factors such as the effect of dissolved electrolytes, laser wavelength, and multiphoton absorption, or developing physics-informed AI methods.^36,95^ For instance, metal linear and nonlinear absorption consistently increase at shorter wavelengths, while the salt concentration and LAL wavelength are known to influence NPs’ size and stability.^9,15,35^ At the same time, this work highlights the positive prospects of ML-optimized LAL for the low-cost and environmentally sustainable production of other NPs made of precious metals and their alloys with convenient properties in terms of surface cleanliness and purity not achievable through wet-chemistry routes.

## Methods

### Synthesis

LAL was conducted under ambient air conditions using an all-fiber RNFL (random nanosecond fiber laser)-seeded MOPA (master oscillator power-amplifier) model P60MX (Raycus Fiber Laser Technologies) and a galvanometric optical scanner (Basicube10, 100 mm aperture, Scanlab). These devices were controlled by the EZCAD software through an industrial standard laser interface driven by a 24 V DC power source. The scanner was equipped with an f-theta lens with a nominal focal length of 100 mm. The scanning pattern allowed for uniform ablation over the target area through Archimedean spirals. The laser output power was measured using a power meter (Gentec-UNO with the detection head UP19K-110F-H9-D0). The synthesis parameters for the training data set are described in Table S1 in S.I. Pyrex cylindrical glass containers (50 mL or 2 L) were filled with Milli-Q water containing 2·10^–4^ M NaCl (Fluka, containing also <0.001% of K). The containers were placed on top of a magnetic mini-stirrer (Microstirrer, Velp), and a Teflon-coated magnetic bar was used to stir the solution during LAL. The target consisted of 99.99% pure Au (a disc with a diameter of 10 mm and 1 mm thickness) supported by a Teflon target holder placed on top of the containers. The gold target was located 3 mm below the liquid level. Before each experiment session, the working distance was varied until the point of maximal productivity, and all the following experiments were performed at this distance.^27^ A microscope slide (3 × 1 in.^2^) was placed between the target and the laser beam to protect the lens from liquid vapors and spills. The glassware, the Teflon holder, the magnetic bar, and the target were washed with Milli-Q water in an ultrasound bath for 15 min before each synthesis. The large batch LAL synthesis was performed for 10 min for productivity experiments or 20 min until reaching a comparable concentration with commercial CS Au NPs.

For LSPR comparison with the CS Au NPs, the LAL Au NPs were centrifuged at 2000 rcf for 1 h, and the supernatant was collected for UV–vis and TEM analysis.

CS Au NPs were purchased from Sigma-Aldrich (5 nm Au NPs, OD 1, stabilized suspension in 0.1 mg/mL citrate buffer, code 741949, batch MKCV7367).

### Characterization

After each LAL synthesis, UV–visible spectra were recorded with a JASCO V770 spectrophotometer by using quartz cells with a 2 mm optical path. In situ concentration analysis for checking the synthesis setup was performed with an Avantes portable spectrometer (AvaSpec-ULS2048CL-EVO) coupled with a deuterium-halogen lamp (AVA-Light-DHc). Au NPs’ concentration and size were measured through the fit of the UV–vis spectrum with the Mie–Gans model, according to a previously published procedure.^47,48^ The gold concentration conversion factor was further validated with ICP-MS analysis. The plasmon damping parameter for the fitting model was calibrated with the actual average Feret size of the Au NPs measured with TEM. DLS and Z-spectroscopy were performed in triplicate with a Malvern Zetasizer Nano ZS in ZEN0112 and DTS1070 cuvettes.

TEM analysis was carried out with an FEI Tecnai G2 12 electron microscope operating at 100 kV and equipped with a TVIPS CCD camera. One drop of the solution was deposited on a copper grid coated with an amorphous carbon film. ImageJ software was used to measure the geometrical (Feret) size distributions, obtained from statistics considering >900 individual Au NPs. HRTEM images were collected with a STEM JEOL JEM F200 instrument operated at 200 kV.

In single-pulse crater analysis, the laser ablation was performed by focusing the laser pulses on a flat bulk gold surface (13 mm × 7 mm × 1 mm thickness, not previously ablated) under 3 mm of water (same as in LAL synthesis) at a scan speed of 8000 mm/s under different laser parameters taken from the experimental data set. Focus was adjusted before single-pulse laser ablation on a distinct portion of the same plate. Scanning electron microscopy (SEM) was performed with a Zeiss Sigma HD field-emission scanning electron microscope (Carl Zeiss, Germany) equipped with a Schottky FEG source. Profilometry was performed with a KLA-Tencor P-17.

### MUA Coating

MUA (Sigma-Aldrich) was dissolved at 1.5 mg/mL in Milli-Q water previously set at pH 9 by the addition of NaOH. Then, the MUA solution was added dropwise under magnetic stirring to the LAL and CS Au NP solutions at 0.1 mg/mL of Au. After mixing, the solutions were sonicated for 10 min and kept overnight before UV–vis analysis. For XPS analysis, the solutions were first dialyzed with concentration membranes (Sartorius, cutoff 10 kDa, 200 RCF, 3 washes with Milli-Q water at pH 9) and then deposited at room temperature by drop casting on glassy carbon substrates. XPS spectra were acquired in an ultrahigh vacuum (UHV) chamber operating at a working pressure below 5 × 10^–8^ mbar. The measurements were performed with Al Kα radiation (hν = 1486.7 eV) with pass energies of 50 eV for survey and B 1s spectra and 20 eV for high-resolution spectra.

### 4-NP Reduction

The pellets of Au NPs were prepared by adding 1 mg of dextran-SH (5 kDa, 5 mol % substitution, HAworks) to 1 mL of LAL or commercial CS Au NPs at a concentration of 2 μg/mL. After 1 h of incubation, the solutions were centrifuged at 10,000 rpm for 1 h. Then, the supernatants were removed and analyzed by UV–Vis spectroscopy at 400 nm, a standard method for determining the concentration of Au NPs in aqueous media.^47,48^ After negligible absorbance (∼0.01) was confirmed in a 2 mm quartz cuvette for both samples, the resulting pellets were added to the 2 mL glass vial (HPLC grade) used as the catalytic reactors. The model catalytic reduction of 4-NP (Sigma-Aldrich) to 4-AP in the presence of NaBH_4_ (Sigma-Aldrich) was carried out in a reactor with a total liquid volume of 1.5 mL, containing 0.15 mM 4-NP and 15 mM NaBH_4_. The large excess of NaBH_4_ relative to that of 4-NP ensured a constant hydrogen donor concentration throughout the reaction.

Three reaction systems were subsequently monitored in a control reactor without Au NPs and in the two reactors containing pellets of LAL and CS Au NPs. The concentration of 4-NP was determined by monitoring its absorbance at 401 nm at 3 min intervals over a total reaction time of 26 min. The reaction kinetics were analyzed using a pseudo-first-order model, expressed as

$$\mathrm{Ln}\left(\frac{A_{t}}{A_{0}}\right)=\mathrm{Ln}\left(\frac{\varepsilon lC_{t}}{\varepsilon lC_{0}}\right)=\mathrm{Ln}\left(\frac{C_{t}}{C_{0}}\right)=-kt$$

where *A*_0_ and *A*_*t*_ are the 4-NP absorbance at time 0 and *t*, respectively, and *C*_*t*_ and *C*_0_ are the corresponding concentrations, proportional to absorbance according to the Beer–Lambert law.^13,67–69^ The constant *k* is the pseudo-first-order kinetic constant.^13,67–69^ The catalytic reduction process was performed in triplicate for each sample to ensure statistical rigor.

### MALDI Experiment

LAL Au NPs were concentrated to 1.3 mg/mL with an Eppendorf Concentrator Plus. CS Au NPs were concentrated to 1.3 mg/mL by dialysis concentration membranes at 500 rcf with a 3000 Da cutoff (Sartorius Vivaspin). The reference organic matrix for low-mass MALDI analysis was α-cyano-4-hydroxycinnamic acid (HCCA, saturated aqueous solution with 0.1% trifluoroacetic acid and 30% acetonitrile). l-Arginine (200 ng/μL in water), l-asparagine (200 ng/μL in water), and atrazine (50 ng/μL in methanol:water 1:1), from Sigma-Aldrich, were used. Each matrix (1.0 μL) and analyte (0.5 μL) were spotted on the standard MALDI stainless steel plate. Mass spectra were recorded with an Ultraflextreme TOF/TOF instrument (Bruker Daltonics) using positive ion detection. LDI was performed with a Smartbeam-II laser set at 27% of the maximum power. Each spectrum was averaged over 2000 laser shots and analyzed with the FlexControl (version 3.4) software. Each analyte was collected within the same measurement session to minimize the possible effects due to fluctuations in spectrometer settings (i.e., laser power) from one session to another or over different days.

### In Vitro Cytotoxicity

LAL Au NPs were concentrated to 2.0 mg/mL with an Eppendorf Concentrator Plus. CS Au NPs were concentrated to 2.0 mg/mL by dialysis concentration membranes at 500 rcf with a 3000 Da cutoff (Sartorius Vivaspin). Human Umbilical Vein Endothelial Cells (HUVECs) and normal human lung fibroblasts (CCD-34Lu) were obtained from the ATCC. HUVECs were cultured in Human Large Vessel Endothelial Cell Basal Medium supplemented with Large Vessel Endothelial Supplement (LVES) (Thermo Fisher Scientific, Milan, Italy), according to the manufacturer’s instructions. CCD-34Lu fibroblasts were maintained in RPMI-1640 Medium, ATCC modification (Thermo Fisher Scientific), supplemented with 10% fetal bovine serum (FBS; Thermo Fisher Scientific). Both cell lines were grown as monolayers in tissue culture flasks and maintained in a humidified incubator at 37 °C with 5% CO2 and 99% relative humidity. The culture medium was refreshed every 2–3 days, and cells were passaged upon reaching 80–90% confluence. The cytotoxicity of LAL and commercial CS Au NPs was assessed using the MTS assay (CellTiter 96 Aqueous One Solution Cell Proliferation Assay, Promega, Milan, Italy) in cells exposed to increasing concentrations (25–200 μg/mL) of AuNPs for 48 h. Cells (8 × 10^3^ cells/well) were seeded in 96-well plates, and after 24 h, the medium was replaced with a fresh one containing AuNPs. For the MTS assay, the medium was replaced with 100 μL of serum-free medium and 20 μL of the CellTiter 96 reagent. After 60–90 min, the absorbance at 492 nm was measured with a Spark plate reader (Tecan, Männedorf, Switzerland), and cell viability was expressed as a function of absorbance relative to that of control cells (considered as 100% viability). The relative dose–response curves were obtained using GraphPad Prism 10.

### ML Regression Analysis

The ANN regression model was applied to the data set of 96 LAL experiments using Python’s scikit-learn (v1.3.2)^96^ and the MLP package. Bayesian optimization coupled with cross-validation was employed for ANN hyperparameter tuning based on R^2^ metric variations.^36–38,96^ The optimal hyperparameter setting of the ANN model is reported in Table S8 of the S.I. Tree-based ensembles (e.g., XGBoost) were also tested as a regression baseline. However, for continuous laser-parameter spaces, stepwise response surfaces over variables such as pulse duration and repetition rate were achieved, with predictions that often plateau within the experimentally sampled range.^49,50^ For this reason, we prioritized the ANN for inverse prediction in our optimization setting, as ANNs typically provide a smoother continuous prediction over the synthesis parameters that better support inverse prediction within a bounded domain.^97^

SHapley Additive Explanations (SHAP) was used to interpret the ANN regression model and quantify the contribution of each synthesis parameter to the predicted Au NP productivity. A SHAP explainer was constructed using the model prediction function with the normalized training feature as background, and SHAP values were computed for the evaluated data set using the SHAP Python package.^50^ Global feature importance was reported as the mean absolute SHAP value across samples, and the distribution and direction of feature effects were visualized by using a SHAP summary (beeswarm) plot with points color-coded by the corresponding normalized feature value. In the summary plot, features are ordered by overall importance (top = highest mean |SHAP|), each point represents one experiment, the *x*-axis position gives the signed impact on the predicted concentration (positive increases, negative decreases), and the color indicates whether that experiment used a relatively low versus high value of the corresponding laser parameter.

### DOE Regression Analysis

DOE’s regression framework was applied through a sequence of increasingly expressive regression forms using the JMP Pro software. The predictive performance of each model was evaluated through its R^2^. These models included a main-effects model (R^2^ = 0.51), a two-way interaction model (R^2^ = 0.76), and a three-way interaction model (R^2^ = 0.80), followed by nonlinear polynomial models including quadratic (R^2^ = 0.72), cubic (R^2^ = 0.76), and partial-cubic (R^2^ = 0.88) regressions derived from all possible combinations of the four original variables (pulse width, repetition rate, scanning speed, and laser power). The partial cubic effects model achieved the best trade-off between accuracy (R^2^ = 0.88) and complexity (root-mean-square error, RMSE = 0.0147), indicating that selective inclusion of higher-order terms captures key nonlinear trends without excessive overfitting. The resulting best-performing regression equation (Figure S3 in S.I.) was then used to identify parameter settings that maximize Au NP productivity (130 ns, 50 kHz, 50 W, 7 m/s).

## Supplementary Material







## Notes

360° rotation of the ML-predicted 3D productivity plot (Movie S1) (MP4)
Parameter table for the experimental data set building (Table S1); productivity trends versus different synthesis parameters in the experimental data set (Figure S1); SHAP dependence plots for main and pairwise interaction effects (Figure S2); DOE model-effect performance comparisons for productivity modeling (Figure S3); comparison of LAL setup cost for this study and benchmark literature results (Figure S4); comparison of productivity with empirical, DOE, and ANN methods (Table S2); violin graph showing the distribution of Au NP sizes in the experimental data set (Figure S5); TEM images and size distributions of CS and sorted LAL Au NPs (Figure S6); XPS survey of MUA-coated LAL and CS Au NP samples (Figure S7); catalytic reduction of 4-NP with NaBH_4_ in the control sample (Figure S8); productivity trends versus pulse energy and other synthesis parameters in the experimental data set (Figure S9); plots of pulse energy versus pulse duration in the experimental data set and in ANN recommendations (Figure S10); plot of thermal penetration depth versus pulse duration (Figure S11); single pulse crater analysis and fluence estimation (Figure S12); evaluation of phase explosion conditions (Table S3); ecoscale evaluation for LAL and CS Au NP synthesis (Table S6); complexGAPI evaluation for LAL and CS Au NP synthesis (Table S7); optimal hyperparameter settings of the ANN model for LAL Au NP synthesis (Table S8) (PDF)
CAPEX and OPEX for LAL Au NPs (Table S4); CAPEX and OPEX for CS Au NPs (Table S5) (XLSX)
