# Supplementary material for: Machine Learning Optimization of Laser Ablation in Liquid for the Green and Low-Cost Synthesis of Clean Gold Nanoparticles
Source: J Am Chem Soc. 2026 Apr 2;148(14):15201–16. doi: 10.1021/jacs.6c02047 (PMC13088233; doi:10.1021/jacs.6c02047)
Supplement: Supplementary file 2 [file ja6c02047_si_002.pdf]

Supporting Information

Machine Learning Optimization of Laser Ablation in Liquid for the Green and Low-Cost Synthesis of Clean Gold Nanoparticles

Runpeng Miao,[1] Catherine Reffatto,[1] Mattia Cattelan,[1] Rafael Torres-Mendieta[1] Luca Menilli,[2] Vincenzo Amendola[1,*]

[1] Department of Chemical Sciences, University of Padova, Via Marzolo 1, Padova I-35131, Italy

[2] Department of Pharmaceutical and Pharmacological Sciences, Via Marzolo 5, Padova I-35131, Italy

* vincenzo.amendola@unipd.it

Contents

**Figure S1**	Productivity trends versus different synthesis parameters in the experimental dataset
**Figure S2**	SHAP dependence plots for main and pairwise interaction effects
**Figure S3**	DOE model-effect performance comparisons for productivity modelling
**Figure S4**	Comparison of LAL setup cost for this study and benchmark literature results
**Figure S5**	Violin graph showing the distribution of Au NPs size in the experimental dataset
**Figure S6**	TEM images and size distributions of CS and sorted LAL Au NPs
**Figure S7**	XPS survey of MUA-coated LAL and CS Au NPs samples
**Figure S8**	Catalytic reduction of 4-NP with $NaBH_4$ in the control sample
**Figure S9**	Productivity trends versus pulse energy and other synthesis parameters in the experimental dataset
**Figure S10**	Plots of pulse energy versus pulse duration in the experimental dataset and in ANN recommendation
**Figure S11**	Plot of thermal penetration depth versus pulse duration
**Figure S12**	Single pulse crater analysis and fluence estimation
**Table S1**	Parameter table for the experimental dataset building
**Table S2**	Comparison of productivity with empirical, DOE and ANN methods
**Table S3**	Evaluation of phase explosion condition
**Table S4**	CAPEX and OPEX for LAL Au NPs (in S.I. file **Tables S4-S5.xlsx**)
**Table S5**	CAPEX and OPEX for CS Au NPs (in S.I. file **Tables S4-S5.xlsx**)
**Table S6**	Ecoscale evaluation for LAL and CS Au NPs synthesis
**Table S7**	ComplexGAPI evaluation for LAL and CS Au NPs synthesis
**Table S8**	Optimal hyperparameter settings of the ANN model for the LAL Au NP synthesis
**Movie S1**	360° rotation of the ML predicted 3D productivity plot (in S.I. file ***Movie S1.mp4***)

Table S1. Parameter table for the experimental dataset building. The 96 parameter combinations distributed within the ranges allowed by the experimental setup used for the collection of the experimental dataset.

Pulse duration (ns)	Repetition rate (kHz)	Laser power	Scanning speed (m/s)
10	250	100% / 20%	7 / 1
	100		
	60		
	20		
20	250		
	100		
	60		
	20		
30	250		
	100		
	60		
	20		
60	250		
	100		
	60		
	20		
100	250		
	100		
	60		
	20		
250	250		
	100		
	60		
	20		

Table S2. Comparison of productivity with empirical, DOE and ANN methods.

Optimization method	mg mL^{-1} min^{-1}	mg min^{-1}	g h^{-1}
Empirical	*0.148 ± 0.017*	*5.92 ± 0.68*	*0.354 ± 0.041*
ANN	*0.193 ± 0.011*	*7.72 ± 0.44*	*0.462 ± 0.025*
DOE	*0.163 ± 0.015*	*6.52 ± 0.60*	*0.392 ± 0.037*

Error represents the standard deviation of at least three replicates.

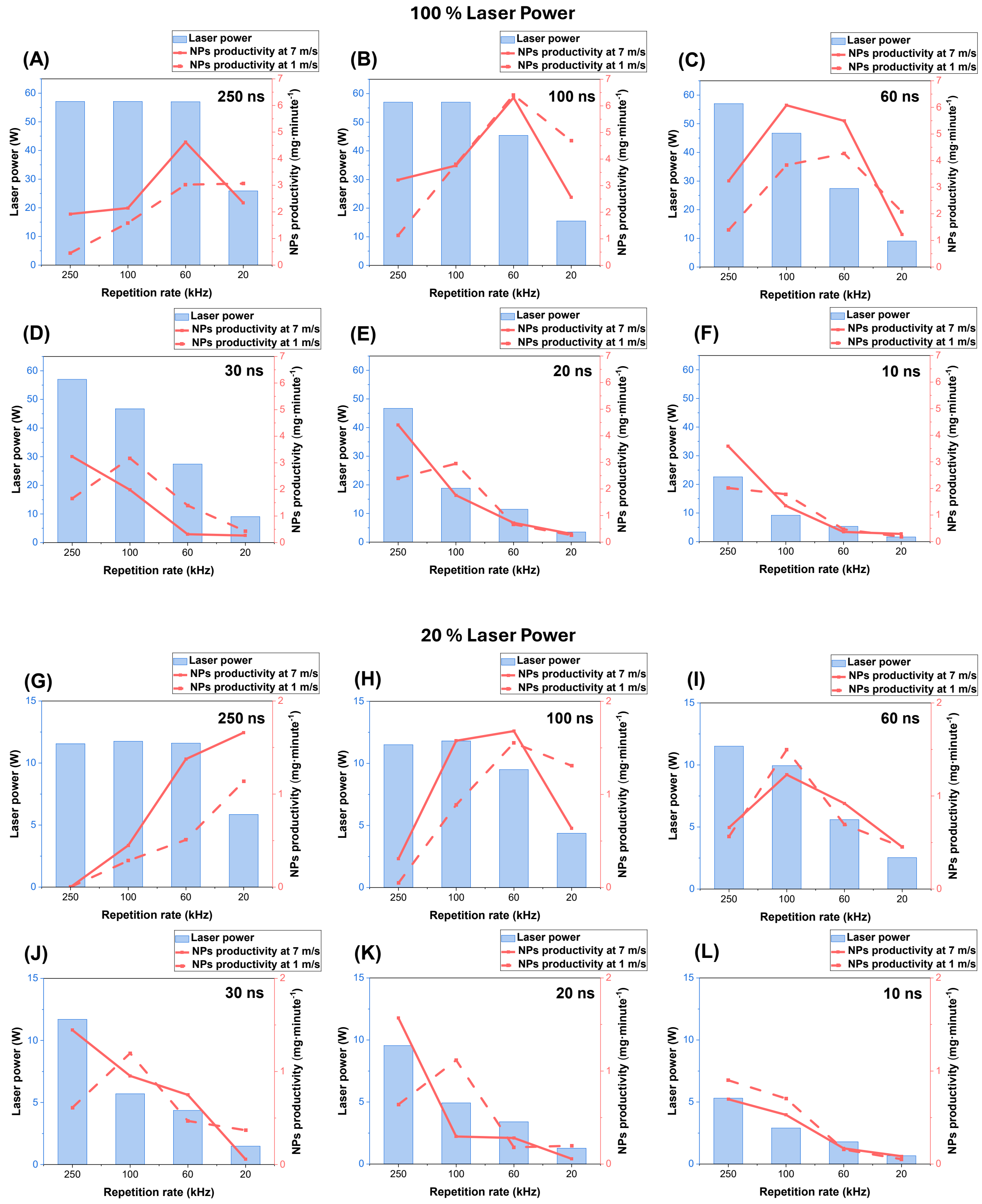

Figure S1. Productivity trends versus different synthesis parameters in the experimental dataset. The analysis of Au NPs productivity as a function of the different synthesis parameters of the experimental dataset, compared with the average laser power. (A-F) The influence of laser repetition rate (250 kHz – 20 kHz), pulse duration (250 ns – 10 ns), and scanning speed (red solid lines: 7 m/s, dashed lines: 1 m/s) on both the average laser power and the productivity of Au NPs under preset experimental conditions. The power is set to 100 % of the laser output at each given condition. (G-L) The influence of laser repetition rate (250 kHz – 20 kHz), pulse duration (250 ns – 10 ns), and scanning speed (red solid lines: 7 m/s, dashed lines: 1 m/s) on both the average laser power and the productivity of Au NPs under preset experimental conditions. The power is set to 20 % of the laser output at each given condition. At both 100% and 20% laser power, the productivity exhibits a nonlinear dependence on both frequency and pulse duration.

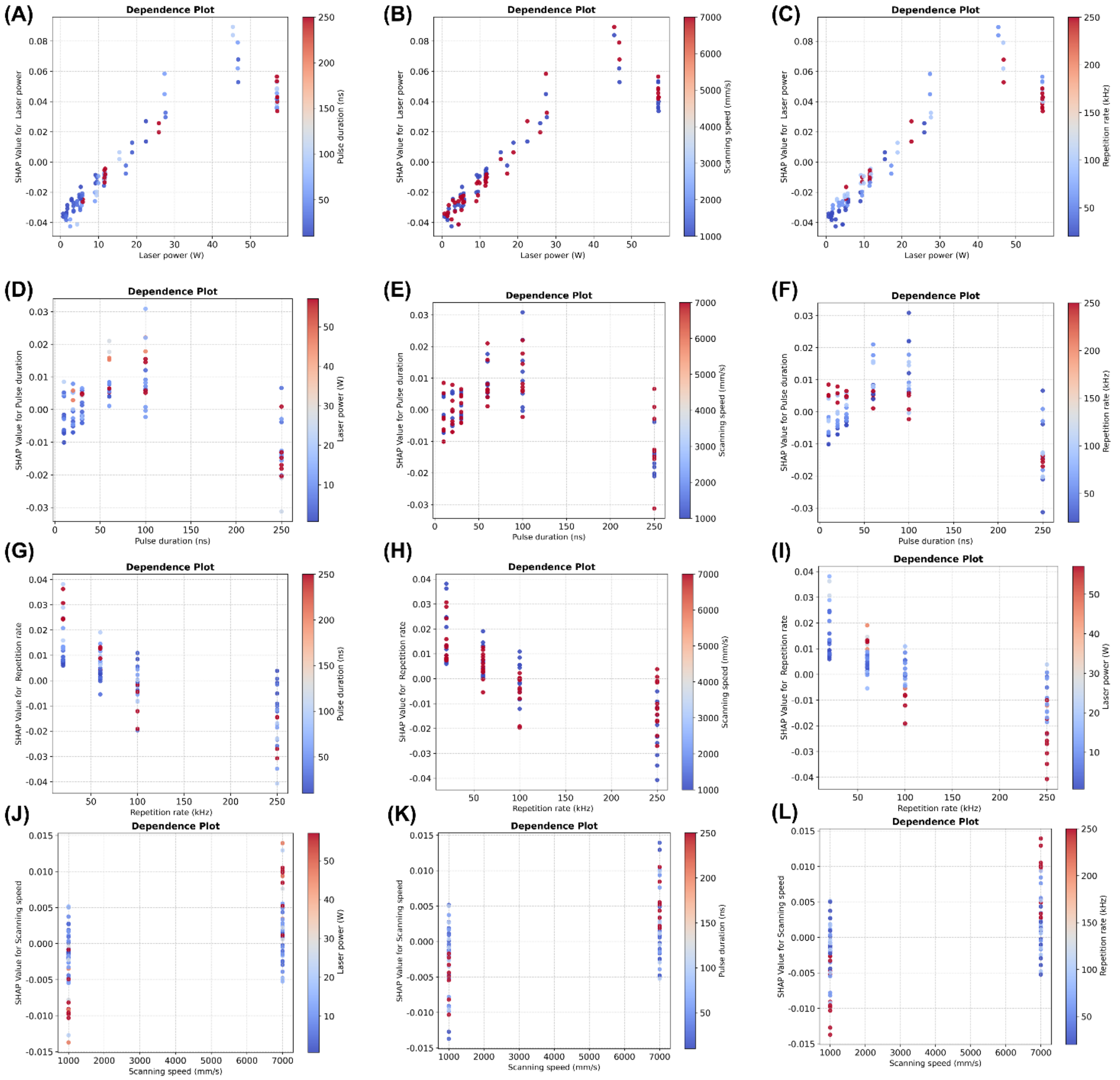

Figure S2. SHAP dependence plots for main and pairwise interaction effects. SHAP dependence plots highlighting main effects and pairwise interactions among laser processing parameters. (A-C) SHAP dependence of laser power (W), coloured by (A) pulse duration (ns), (B) scanning speed (mm·s^{-1}) and (C) repetition rate (kHz). (D-F) SHAP dependence of pulse duration (ns), coloured by (D) laser power (W), (E) scanning speed (mm·s^{-1}) and (F) repetition rate (kHz). (G-I) SHAP dependence of repetition rate (kHz), coloured by (G) pulse duration (ns), (H) scanning speed (mm·s^{-1}) and (I) laser power (W). (J-L) SHAP dependence of scanning speed (mm·s^{-1}), coloured by (J) laser power (W), (K) pulse duration (ns) and (L) repetition rate (kHz). Each point corresponds to one experiment; the x-axis reports the feature value, and the y-axis reports the corresponding SHAP value (marginal contribution relative to the model baseline). Colour encodes the interacting feature to visualize non-linearities and interaction patterns. Positive (negative) SHAP values indicate an increase (decrease) in the predicted response.

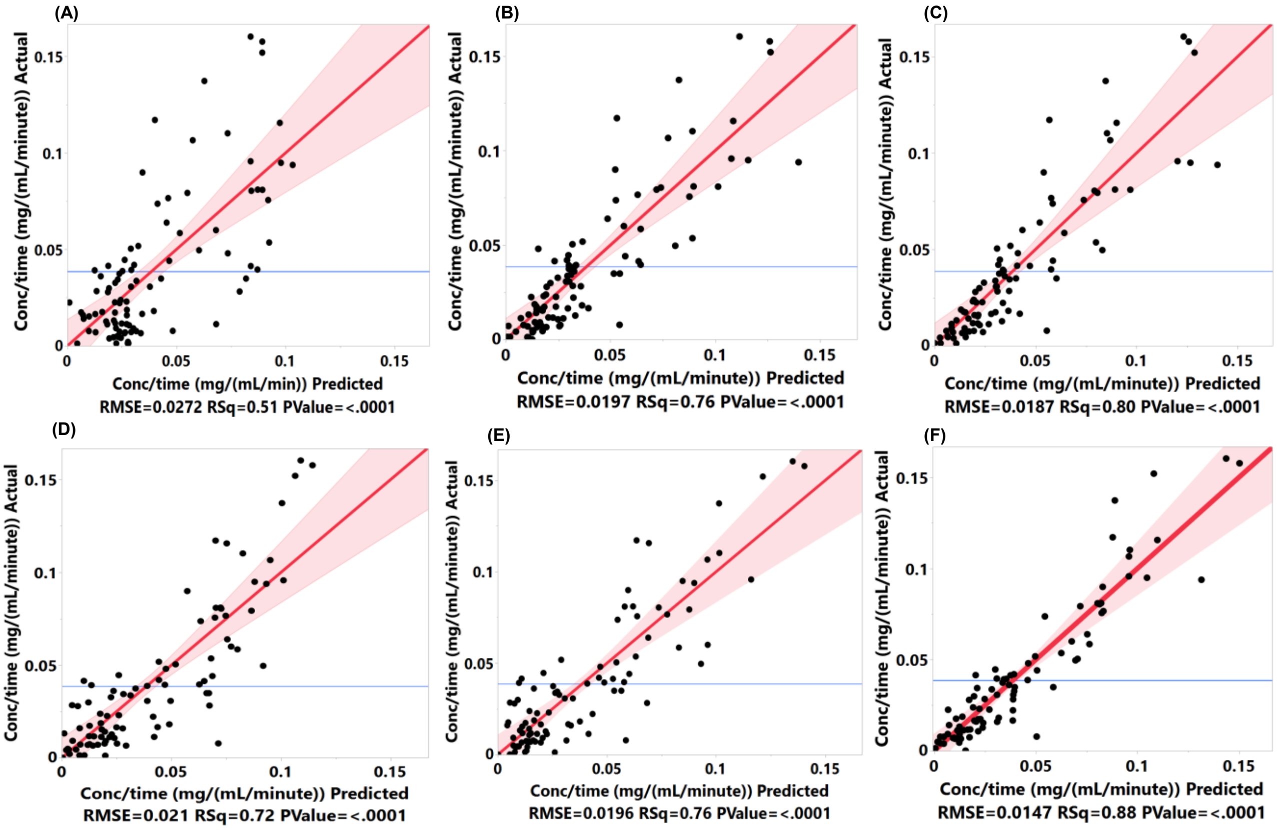

Figure S3. DOE model-effect performance comparisons for productivity modelling. DOE fitting performance comparison between different constructed model effects for modelling the NPs productivity and laser processing parameters. (A) main effects; (B) two-way interaction effects; (C) three-way interaction effects; (D) quadratic effects; (E) cubic effects; (F) partial cubic effects. The main effects model, which considers only the independent contributions of each laser parameter, exhibits the lowest predictive accuracy ($R^2 = 0.51$), indicating that single-factor analysis is insufficient to describe the complex interactions governing NP productivity. By incorporating two-way interactions, the predictive capability improves significantly ($R^2 = 0.76$), demonstrating that synergistic effects between laser parameters play a crucial role. Extending this to three-way interactions further refines the prediction ($R^2 = 0.80$), suggesting that higher-order parameter dependencies contribute meaningfully to productivity variations. Nonlinear regression approaches reveal additional insights into the system's complexity. The quadratic model captures second-order effects ($R^2 = 0.72$), though its improvement over the two-way interaction model remains limited. Introducing cubic terms enhances the model's ability to describe more intricate parameter dependencies ($R^2 = 0.76$), yet the performance gain is modest, indicating potential overfitting when higher-order terms are introduced indiscriminately. Among all tested models, the partial cubic effects model demonstrates the highest predictive accuracy ($R^2 = 0.88$) with the lowest RMSE (0.0147).

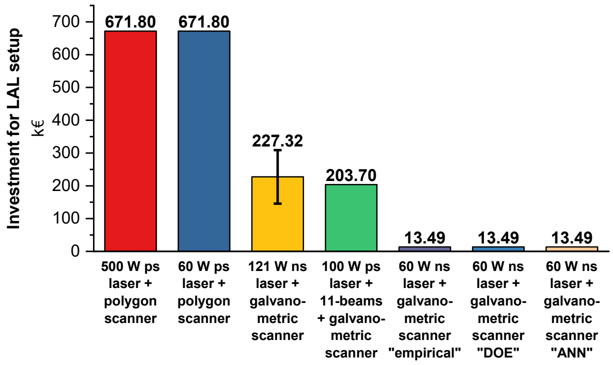

Figure S4. Comparison of setup cost for this study and other benchmark LAL Au NPs synthesis systems. Cost of the 500 W ps laser + polygon scanner, 60 W ps laser + polygon scanner and 100 W ps laser + 11-beams + galvanometric scanner are taken from ref.[1]. The cost of 121 W ns laser + galvanometric scanner[2] is taken from the average of two quotations, one for raw components without import and installation expenses and one for the assembled system including importation and installation, both dated March 2026. The cost of the setup for this study is taken from the invoice of purchase by the Authors.

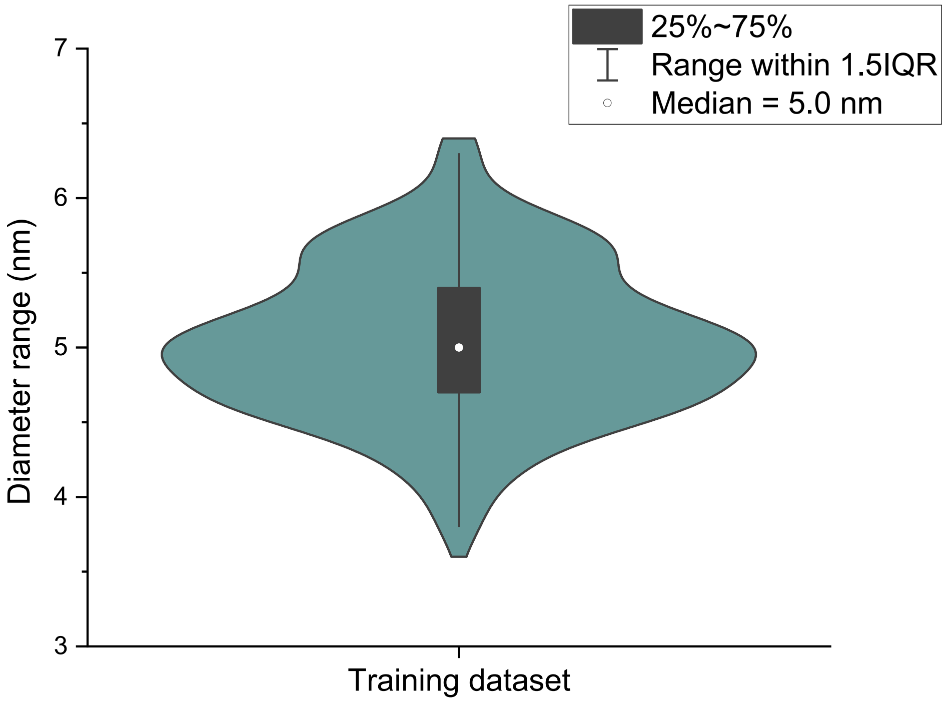

Figure S5. Violin graph showing the distribution of Au NPs size in the experimental dataset. NP size is evaluated with the Mie-Gans model fitting (see Methods), calibrated with independent TEM measurement of geometrical size in selected samples. The bar represents 1.5 times the interquartile range (IQR).

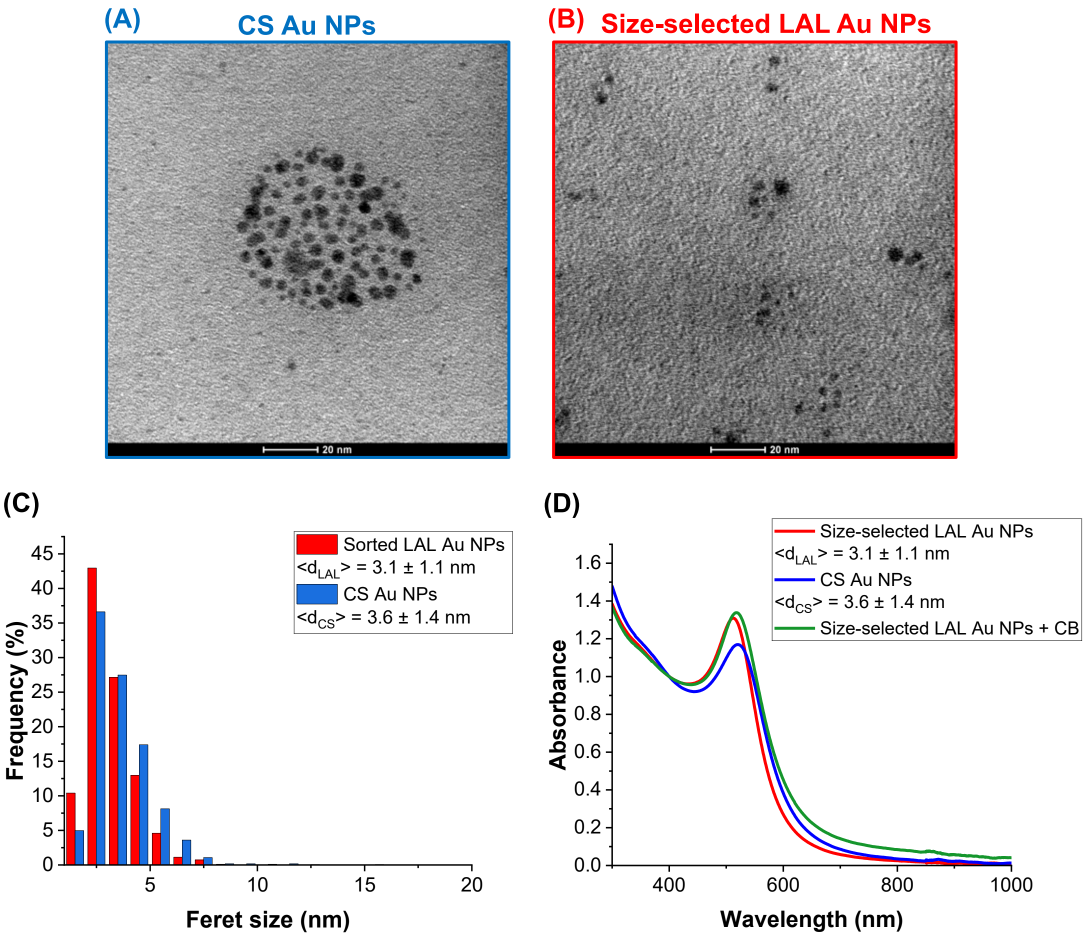

Figure S6. TEM images (A-B) and size distribution (C) of commercial CS Au NPs (N > 1500) and of LAL Au NPs (N > 900) size-selected with a selective sedimentation protocol to achieve comparable average geometrical size. (D) Comparison of UV-vis spectra of the size-selected LAL (red line) and CS Au NPs (blue line). The spectrum of the size-selected LAL Au NPs after addition of citrate buffer at 0.1 mg/mL final concentration, as in the CS Au NPs, is also shown (green line).

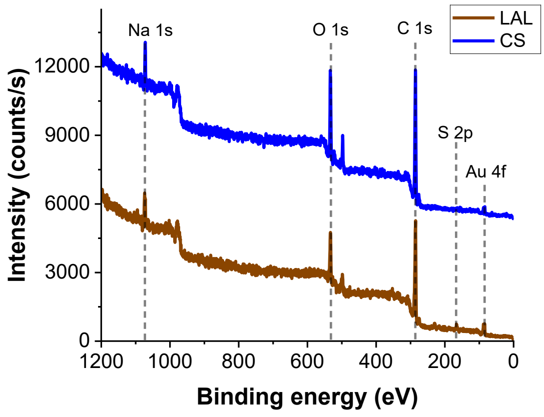

Figure S7. XPS surveys of MUA-coated LAL (black) and CS (blue) Au NPs samples. Higher C 1s and O 1s peak intensities but lower S 2p intensities relative to Au 4f are found in the CS sample, compared to the LAL. The additional C and O are compatible with the persistence of citrate molecules in the CS Au NPs.

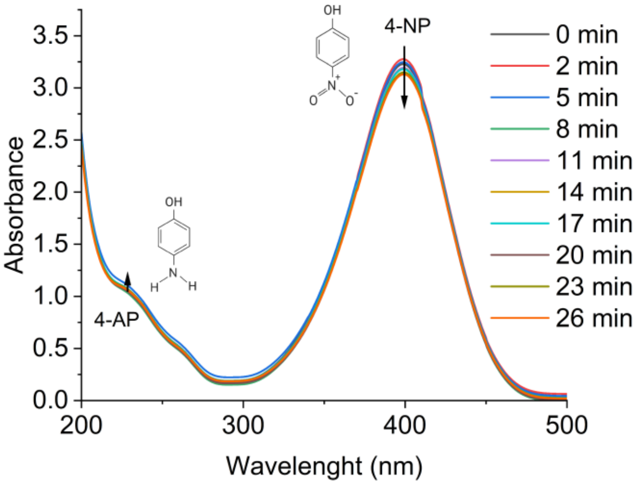

Figure S8. Catalytic reduction of 4-NP with $NaBH_4$ in the control sample. The conversion is indicated by the reduction of the 4-NP band at 400 nm, while that of 4-AP at 230 nm increases.

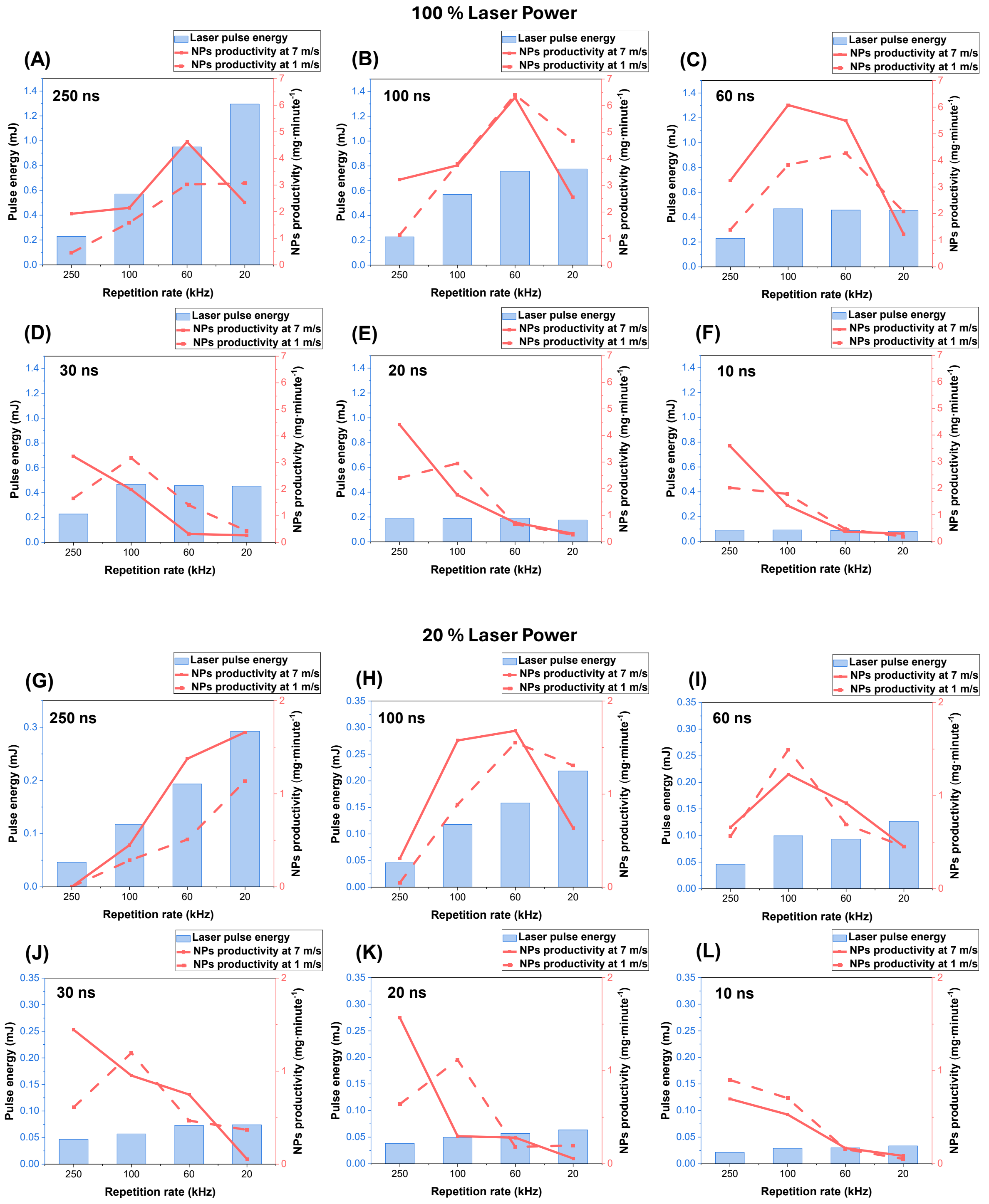

Figure S9. Productivity trends versus pulse energy and other synthesis parameters in the experimental dataset. The analysis of Au NPs productivity as a function of the different synthesis parameters of the experimental dataset, compared with the average single-pulse laser energy. (A-F) The influence of laser repetition rate (250 kHz – 20 kHz), pulse duration (250 ns – 10 ns), and scanning speed (red solid lines: 7 m/s, dashed lines: 1 m/s) on both the average single-pulse laser energy and the productivity of Au NPs under preset experimental conditions. The power is set to 100 % of the laser output at each given condition. (G-L) The influence of laser repetition rate (250 kHz – 20 kHz), pulse duration (250 ns – 10 ns), and scanning speed (red solid lines: 7 m/s, dashed lines: 1 m/s) on both the average single-pulse laser energy and the productivity of Au NPs under preset experimental conditions. The power is set to 20 % of the laser output at each given condition. At both 100% and 20% laser power, the productivity exhibits a nonlinear dependence on both frequency and pulse duration.

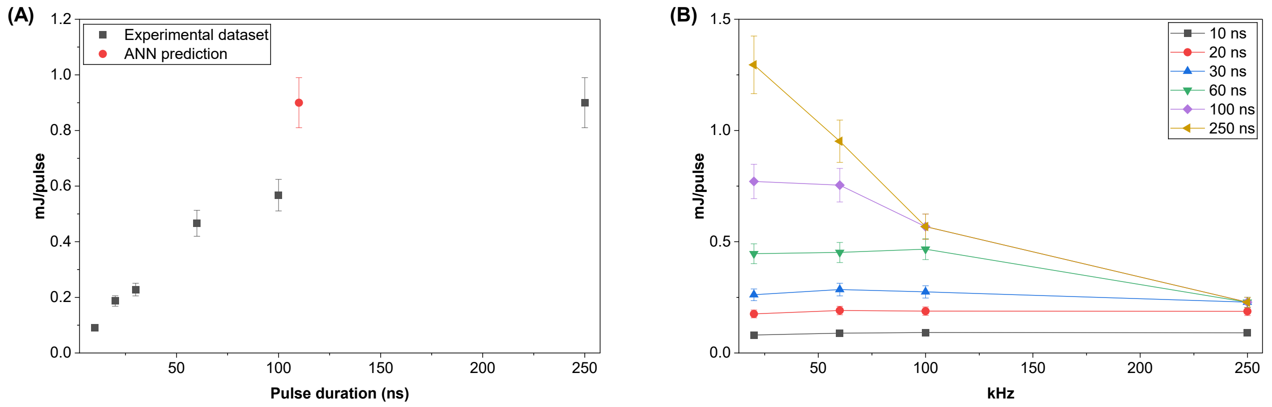

Figure S10. Plots of pulse energy versus pulse duration in the experimental dataset and in ANN recommendation. (A) Plot of energy per laser pulse versus pulse duration at the condition with highest productivity in the experimental dataset (black squares) and for the ANN prediction (red circle). The plot shows that highest productivity, observed at 100 ns for the experimental dataset and at 110 ns for the ANN prediction, does not correspond with highest laser pulse energy. (B) Plot of energy per laser pulse versus repetition rate at different pulse durations in the experimental dataset, showing that higher pulse energy is achieved at lower frequency for 100 and 250 ns, whereas pulse energy remains comparable for 30, 20 and 10 ns, with only a moderate increment at low repetition rate for the 60 ns.

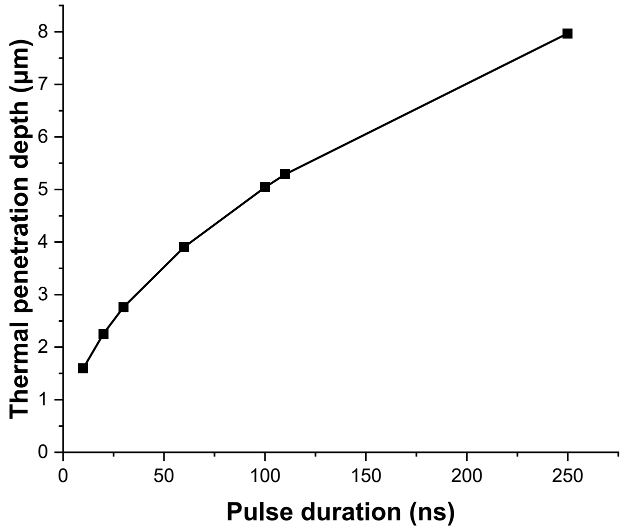

Figure S11. Plot of thermal penetration depth (L_{th}) versus pulse duration (τ), calculated according to $L_{th} = (2\delta\tau)^{1/2}$, where δ is Au thermal diffusivity.[3]

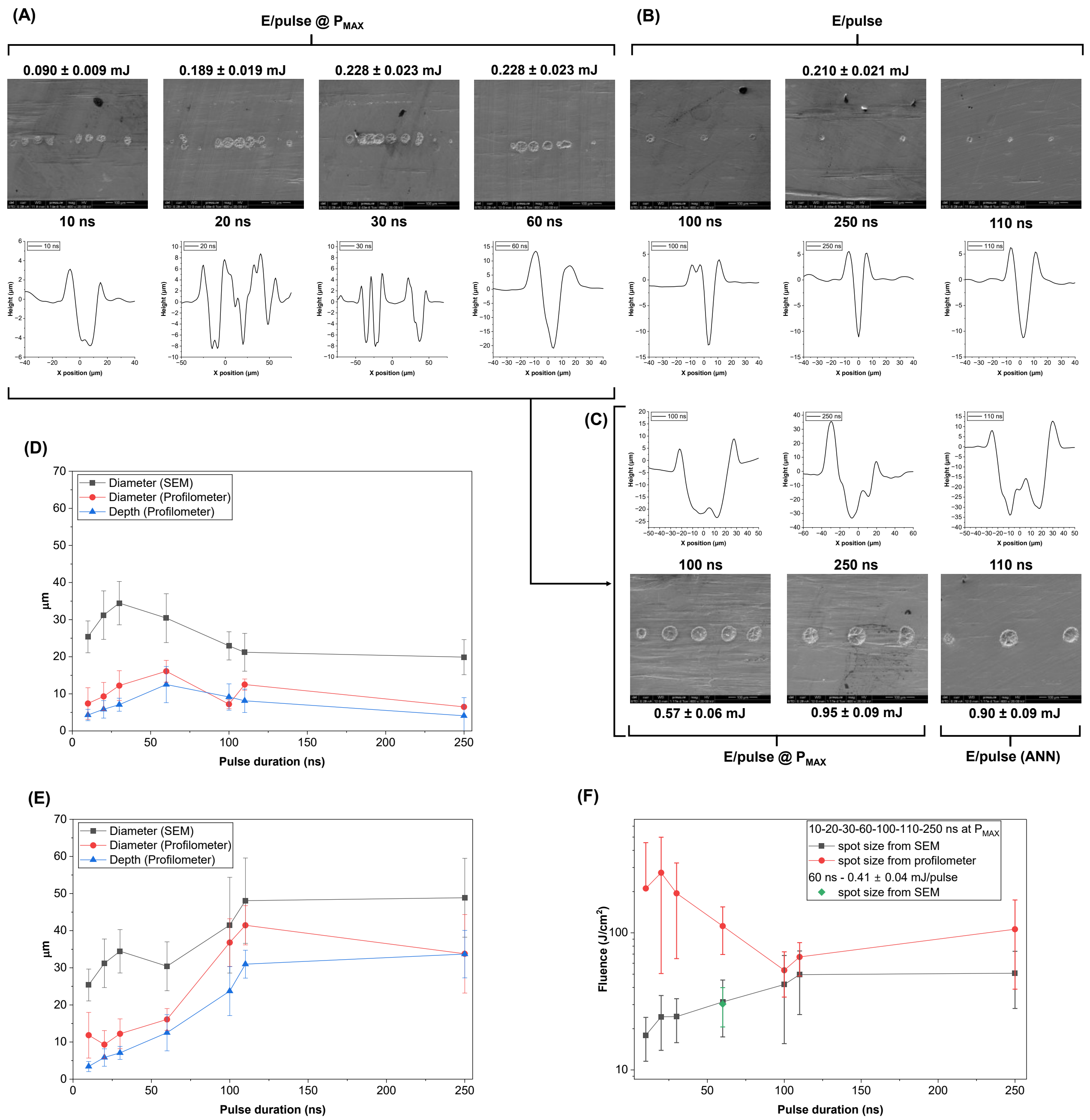

Figure S12. Single pulse crater analysis and fluence estimation. (A-C) Representative SEM images and profilometer linescans of single-pulse craters obtained on a flat bulk gold surface (13 mm × 7 mm × 1 mm thickness, not previously ablated) under 3 mm of water at a scan speed of 8000 mm/s under different laser parameters. For pulse durations of 10, 20, 30 and 60 ns (A), the laser parameters correspond to those with highest output power (P_{MAX}) in the experimental dataset for the corresponding pulse duration. For 100 and 250 ns, the experiment was performed at the laser parameters at P_{MAX} and highest productivity in the experimental dataset (C), and then repeated after decreasing the energy per pulse (E/pulse) to the same value as the 20, 30 and 60 ns pulses (B). For 110 ns (B-C), both the parameters of ANN recommendation and E/pulse equal to 20, 30 and 60 ns were tested. Note that SEM and profilometer experiments were performed in distinct measurement sessions, therefore SEM images and profilometer linescans do not refer to the same craters. (D) Plot of average single-pulse crater diameters measured by SEM (black dots) and profilometer (red circles) and average crater depths measured with profilometer (blue triangles) for the experiment with comparable E/pulse among 20, 30, 60, 100, 250 and 110 ns pulses. (E) Plot of average single-pulse crater diameters measured by SEM (black dots) and profilometer (red circles) and average crater depths measured with profilometer (blue triangles) for the experiment at P_{MAX} in the experimental dataset for 10, 20, 30, 60, 100, 250, and ANN recommendation for 110 ns pulses. In (D-E), error bars are the standard deviation on a minimum of 3 craters

for profilometer analysis and 6 craters for SEM. (F) Fluence calculated from SEM (black squares) and profilometer (red circles) spot diameters. For the 60 ns pulses, the highest productivity condition in the experimental dataset was also included (green diamond for SEM diameter).

Table S3. Evaluation of phase explosion condition. Phase explosion is generally attributed to homogeneous nucleation within a metastable superheated melt as the transient lattice temperature approaches approximately 0.8–0.9 T_c (phase explosion threshold temperature), which is used as a practical criterion for homogeneous bubble nucleation in a superheated melt, i.e., the onset of "phase explosion."[3,4] For Au, Kalus et al. give T_c ≈7400 K, so 0.8–0.9 ≈ 5900-6600 K.[1] According to the thermodynamic model of phase explosion proposed by Miotello and Kelly,[5,6] the threshold fluence F_{th} can be estimated by considering the energy required to heat the thermal diffusion layer to the critical temperature T_{tc} = 0.8–0.9 T_c:

$F_{th} = \frac{\rho C_p (T_{tc} - T_0) L_{th}}{1-R} = 27.6\ \text{J/cm}^2$	
$\triangle T = (T_{tc} - T_0) \cdot T_{tc} = 0.90\ T_c$	6600 K
T_0 (ambient temperature)	300 K
L_{th} (thermal penetration depth of Au for τ = 110 ns)	5.29 μm
1-R (Au surface absorptivity under liquid[7])	0.3
ρ (density of Au)	19300 kg/m^3
C_p (specific heat of Au)	129 J/(Kg·K)

Tables S6. Ecoscale[8] evaluation for LAL (A) and CS (B) Au NPs synthesis, according to the ecoscale calculator (https://ecoscale.cheminfo.org/)

(A) LAL Au NPs synthesis

Reagents

☑ Link

	identifier*	name	MF*	MW	density	purity*	ml	g	mmoles	equiv.
1		Gold	Au	196.9665		99.99%	0	1.001	5.0815742778	1
2		Sodium chloride	ClNa	58.44277		99.9%	0	0.0021	0.0358966558	0.0070640816

Products

identifier*:	name:	MF*:	MW:	g:	mmoles:	g theor:	yield:
	Gold	Au	196.9665	1	5.0770054806	1.0009	99.9101

Conditions

Reagents

Name	mmoles	eq.	Bp	Hazard	Price
Gold	5.08	1	2800		
Sodium chloride	0.03	0	2800		

	Possible items	Selected items	
Yield	100		0
Price / availability			-5
Safety			-5
Technical setup	Common set-up; Instruments for controlled addition of chemicals; Unconventional activation technique	Common set-up	0
Temperature / time	Room temperature, < 1h; Room temperature, < 24h; Heating, < 1h	Room temperature, < 24h	-1
Workup and purification	None; Cooling to room temperature; Adding solvent	None	0
**EcoScale**			89

(B) CS Au NPs synthesis

Reagents

☑ Link

	identifier*	name	MF*	MW	density	purity*	ml	g	mmoles	equiv.
1		Hydrogen tetrachloroaurate(III)trihydrate	HAuCl4 . 3H2O	393.83228		99.99%	0	1.999689	5.0770054806	1
2		Citric acidtrisodium salt dihydrate	C6H5Na3O7 . 2I	294.10137	1.76	100%	4.238636	7.46	25.365403772	4.9961348020

Products

identifier*:	name:	MF*:	MW:	g:	mmoles:	g theor:	yield:
	Gold	Au	196.9665	1	5.0770054806	1	100

Conditions

Reagents

Name	mmoles	eq.	Bp	Hazard	Price
Hydrogen tetrachloroaurate(III)trihydrate	5.07	1	2800		
Citric acidtrisodium salt dihydrate	25.36	4.99			

	Possible items	Selected items	
Yield	100		0
Price / availability			-5
Safety			-5
Technical setup	Common set-up; Instruments for controlled addition of chemicals; Unconventional activation technique	Common set-up	0
Temperature / time	Room temperature, < 1h; Room temperature, < 24h; Heating, < 1h	Heating, < 1h	-2
Workup and purification	Sublimation; Liquid - liquid extraction or washing; Classical chromatography	Liquid - liquid extraction or washing	-3
**EcoScale**			85

Tables S7. ComplexGAPI[9] evaluation for LAL (A) and CS (B) Au NPs synthesis, considering the different sample preparation methods followed by UV-vis analysis of the results.

(A) LAL Au NPs synthesis

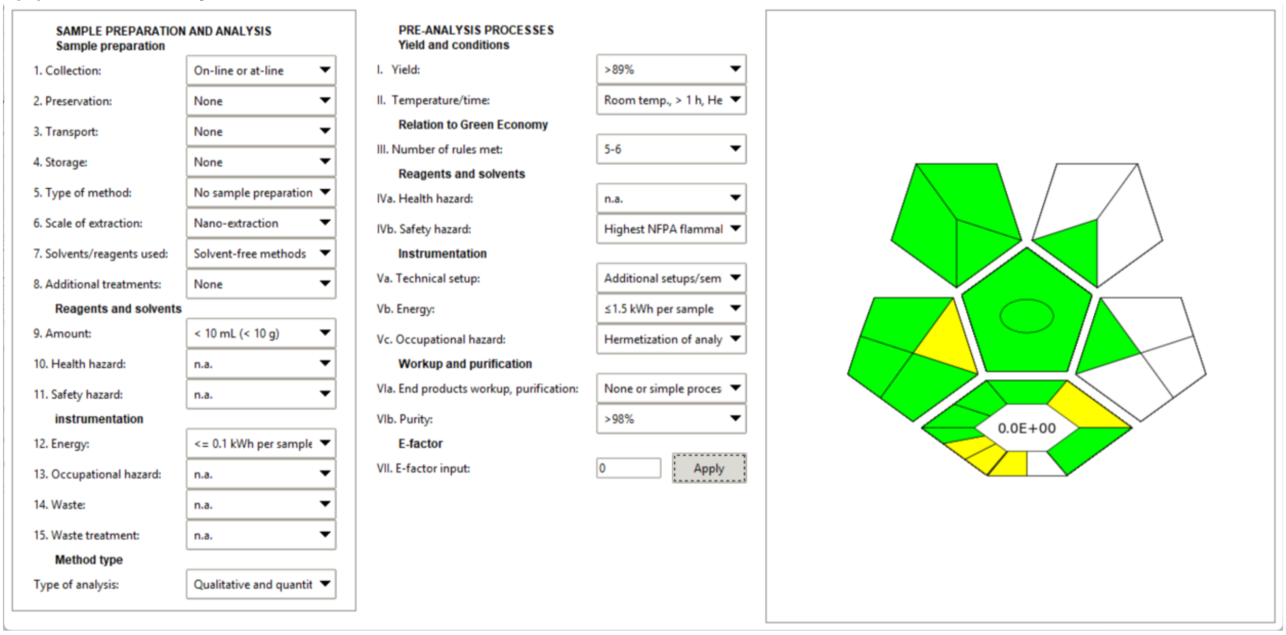

(B) CS Au NPs synthesis

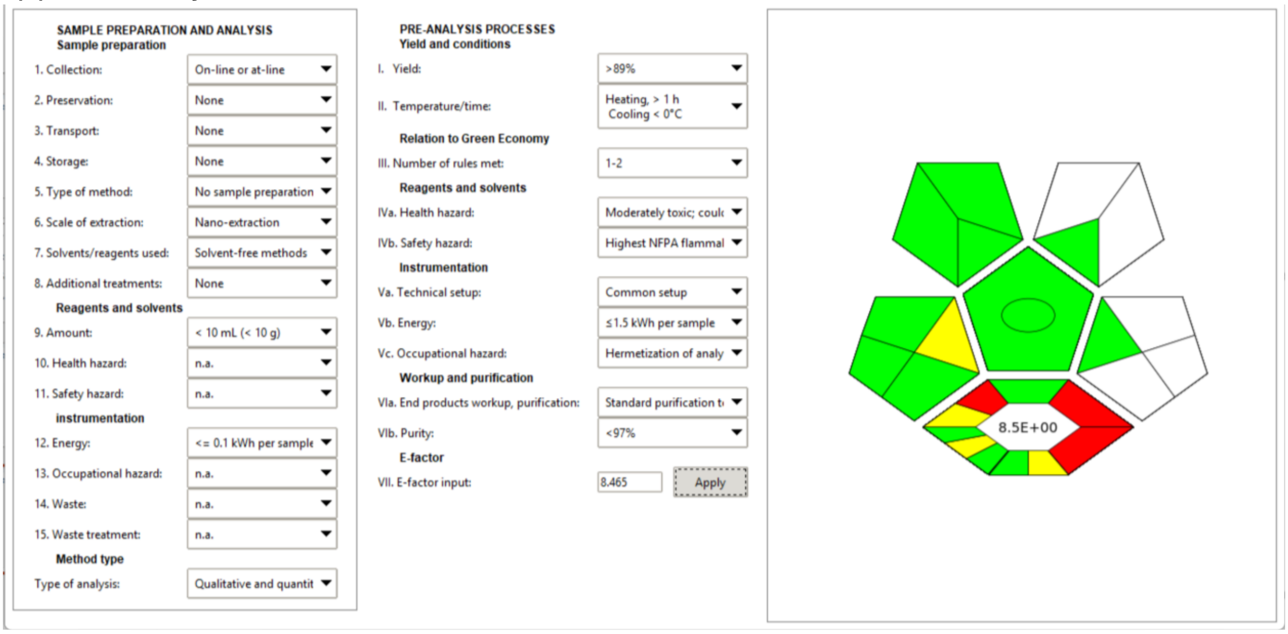

Table S8. Optimal hyperparameter settings of the ANN model for the LAL Au NP synthesis.

Hyperparameter	MLP regressor (ANN model)
**Number of hidden layers**	3
**Neurons of each hidden layer**	(42, 98, 9)
**Activation**	Relu
**Solver**	L-BFGS
**Learning_rate_init**	0.8308556818665724
**Batch size**	5
**Random state**	3804
**Validation fraction**	0.2
**Alpha**	1e-05
**Tol**	0.00041
**Max_fun**	14184

Bibliography.

(1) Khairani, I. Y.; Spellauge, M.; Riahi, F.; Huber, H. P.; Gökce, B.; Doñate-Buendía, C. Parallel Diffractive Multi-Beam Pulsed-Laser Ablation in Liquids Toward Cost-Effective Gram Per Hour Nanoparticle Productivity. *Adv. Photonics Res.* **2024**, *5* (5), 2300290. https://doi.org/10.1002/adpr.202300290.

(2) Kohsakowski, S.; Seiser, F.; Wiederrecht, J. P.; Reichenberger, S.; Vinnay, T.; Barcikowski, S.; Marzun, G. Effective Size Separation of Laser-Generated, Surfactant-Free Nanoparticles by Continuous Centrifugation. *Nanotechnology* **2020**, *31* (9), 095603. https://doi.org/10.1088/1361-6528/ab55bd.

(3) Kalus, M. R.; Barcikowski, S.; Gökce, B. How the Physicochemical Properties of the Bulk Material Affect the Ablation Crater Profile, Mass Balance, and Bubble Dynamics During Single-Pulse, Nanosecond Laser Ablation in Water. *Chem. – A Eur. J.* **2021**, *27* (19), 5978–5991. https://doi.org/10.1002/CHEM.202005087.

(4) Xu, X. Phase Explosion and Its Time Lag in Nanosecond Laser Ablation. In *Applied Surface Science*; 2002; Vol. 197–198, pp 61–66. https://doi.org/10.1016/S0169-4332(02)00304-5.

(5) Miotello, A.; Kelly, R. Critical Assessment of Thermal Models for Laser Sputtering at High Fluences. *Appl. Phys. Lett.* **1995**, *67*, 3535. https://doi.org/10.1063/1.114912.

(6) Bulgakova, N. M.; Bulgakov, A. V. Pulsed Laser Ablation of Solids: Transition from Normal Vaporization to Phase Explosion. *Appl. Phys. A Mater. Sci. Process.* **2001**, *73* (2), 199–208. https://doi.org/10.1007/s003390000686.

(7) Marla, D.; Bhandarkar, U. V.; Joshi, S. S. A Model of Laser Ablation with Temperature-Dependent Material Properties, Vaporization, Phase Explosion and Plasma Shielding. *Appl. Phys. A Mater. Sci. Process.* **2014**, *116* (1), 273–285. https://doi.org/10.1007/s00339-013-8118-0.

(8) Van Aken, K.; Strekowski, L.; Patiny, L. EcoScale, a Semi-Quantitative Tool to Select an Organic Preparation Based on Economical and Ecological Parameters. *Beilstein J. Org. Chem.* **2006**, *2*. https://doi.org/10.1186/1860-5397-2-3.

(9) Płotka-Wasylka, J.; Wojnowski, W. Complementary Green Analytical Procedure Index (ComplexGAPI) and Software. *Green Chem.* **2021**, *23* (21), 8657–8665. https://doi.org/10.1039/d1gc02318g.
